# The effect of *seasonality* in predicting the level of crime. A spatial perspective

**DOI:** 10.1371/journal.pone.0285727

**Published:** 2023-05-31

**Authors:** Rosario Delgado, Héctor Sánchez-Delgado

**Affiliations:** 1 Department of Mathematics, Universitat Autonoma de Barcelona, Cerdanyola del Valles, Spain; 2 Data Quality and Statistics Executive at Kantar TSN, Sant Cugat del Valles, Spain; Universidade Estadual de Maringa, BRAZIL

## Abstract

This paper presents an innovative methodology to study the application of *seasonality* (the existence of cyclical patterns) to help predict the level of crime. This methodology combines the simplicity of entropy-based metrics that describe temporal patterns of a phenomenon, on the one hand, and the predictive power of machine learning on the other. First, the classical Colwell’s metrics *Predictability* and *Contingency* are used to measure different aspects of *seasonality* in a geographical unit. Second, if those metrics turn out to be significantly different from zero, supervised machine learning classification algorithms are built, validated and compared, to predict the level of crime based on the time unit. The methodology is applied to a case study in Barcelona (Spain), with *month* as the unit of time, and *municipal district* as the geographical unit, the city being divided into 10 of them, from a set of property crime data covering the period 2010-2018. The results show that (a) Colwell’s metrics are significantly different from zero in all municipal districts, (b) the month of the year is a good predictor of the level of crime, and (c) Naive Bayes is the most competitive classifier, among those who have been tested. The districts can be ordered using the Naive Bayes, based on the strength of the month as a predictor for each of them. Surprisingly, this order coincides with that obtained using *Contingency*. This fact is very revealing, given the apparent disconnection between entropy-based metrics and machine learning classifiers.

## Introduction

It is commonly accepted that most types of crime show seasonal fluctuations (albeit with differences between them). By *seasonality* is meant a cyclical pattern that repeats itself at regular intervals. *Seasonality* and temporal patterns in crime have been studied since the mid-19th century and continue to be so today (see [1] and references therein). One of the pioneers of studies on *seasonality* in crime was Adolphe Quételet, author of the Thermal Laws of Delinquency in his famous work *Sur l’homme et le développement de ses facultés, ou Essai de physique sociale* (1835), which stated that different types of crime are influenced by gender, age, sex, education, and climate [2, 3]. In relation to the weather, he affirmed that crimes against property were more frequent in winter than in summer while, on the contrary, crimes against people were more frequent in summer due to the temperature and the more lively human passions, and the sexual offenses were more frequent in spring, the breeding season for most species [4]. By contrast, most of the work that has found *seasonality* since the early 20th century, has found summer crime peaks in aggregated offenses such as crimes against property or violent crimes.

In this sense, the authors of [5] suggested that seasonal fluctuations affect crime not only based on the primitive instincts of the individual, but also in the behaviour patterns of society. In fact, there are changes in usual activities during different periods of the year depending on the temperature, and a consequent increase or decrease in the risk of victimization. As Cohen and Felson’s Theory of Routine Activities explains, three elements must be present for a crime to occur: a motivated offender, a suitable target, and the absence of guards or surveillance. The Theory of Routine Activities is part of the Ecological Theories of Crime, which are based on the ecosystems of nature [6]. These theories study the influence of the environment on criminal activity in a giving place, and suggest that there are places that, regardless of who inhabits them, house some human organizations that produce a greater number of crimes, or more serious crimes, than others. They are based on the idea that environmental conditions create an opportunity to commit a crime. The three elements of Routine Activities vary with the seasons and can explain criminal variations. For example, in [7] a positive correlation in found between the deviations of the number of crimes from the expected value, on the one hand, and the temperature deviations from the monthly average, on the other, for almost all types of crime. In addition, the author explains that a higher temperature implies a greater amount of time away from home and a greater risk of being a victim of a robbery with force (since the house is left unprotected) or a robbery, since the outside environment facilitates it.

Currently, thanks to technical improvements that allow accessing and analyzing large data sets that contain geographic information, it is possible to introduce methodologies that allow these statements to be contrasted, taking into account the interaction between spatial and temporal aspects that could contribute to the emergence of criminal opportunities, carrying out an updated review of the phenomenon of crime *seasonality* more in line with the reality of modern society and the possibilities of a scientific approach.

### Motivation

The first approach to the study of the phenomenon of *seasonality* of crime has been the attempt to explain it through the Theory of the Temperature-Aggression Relation. This theory assumes a positive correlation between aggression-inducing irritability and rising temperatures, and predicts a consequent increase in criminal violence in the summer (in the northern hemisphere). However, this theory cannot explain other fluctuations in violent crime or seasonal fluctuations in property crime, which is non-violent. Indeed, some reputable studies, such as [8], observe significant monthly differences in the crime rate, for both violent crime and property crime, regardless of temperature differences, even in different geographical areas with similar temperatures, so this theory might not satisfactorily explain the *seasonality* of crime.

The Theory of Routine Activities, on the other hand, seems to offer a more complete approximation to the *seasonality* of crime, being able to explain it both in violent crimes and in crimes against property, based on changes in the environment that modify the activities of potential victims and can influence their contact with criminals. In fact, the activities of daily life in the city of Barcelona (Spain), which is the case study here, have changed drastically since the 1990s and the 1992 Olympic Games. At that time, the seaside neighborhood named “Barceloneta” was transformed into a space oriented towards the beach, the city opened up to tourism, improved its infrastructures, and became known to the world. In 1990 Barcelona had about two million visitors, in 2013 this number increased to 7.5 million and in 2018 to 8 million. Given the importance of tourism in this city, which has a strong seasonal component, it is undeniable that there has been a change in the routine activities of its inhabitants, and this could be an explanation for the seasonal nature of crime.

But it could also be the case that changes in routine activities were a mediating factor between the weather and the level of crime, in such a way that temperature does affect the level of crime through the changes it induces in the routine activities of the victims, and not so much by directly affecting the psychology of criminals. In this way, the Theory of Routine Activities would modify the sense in which the Theory of the Temperature-Aggression Relation explains the *seasonality* of crime.

Given the complexity of human behavior and the inevitable presence of noise in crime data, reducing the explanation of the *seasonality* phenomenon to one of these theories is undoubtedly an oversimplification. The only thing that seems to be clear is that the reasons for the *seasonality* of crime, if any, are not easy to explain or fit into a theory. Data can reveal correlations and trends, but not their cause or explanation. In this context of uncertainty, the motivation of this research is to approach the phenomenon of the *seasonality* of crime from a pragmatic point of view, giving up trying to explain it so as not to fall into interpretive biases, but instead trying to detect, describe and even predict it quantitatively, following a data-based approach.

Considering that the interest of this research is to discover temporal patterns when addressing the *seasonality* of crime, if they exist, it is natural to wonder whether *Predictability* and its component measures, introduced in [9] and used to describe temporal patterns of biological phenomena in animal and plant ecology, could be successfully applied in this social context. In an attempt to clarify this issue, an in-depth study of crime *seasonality* in the city of Barcelona has been carried out, which encompasses geographical units with different characteristics and with highly diverse crime rates. And this leads to approach this research from a spatial perspective, which would be in accordance with the hypothesis pointed out in [10] that crime is not evenly distributed in a city, but rather is naturally organized into five concentric circular zones, and that the central part (called *Transition Zone*) is where crime is most concentrated, which means that some parts of the city are more crime-ridden than others [11].

### Goals and tools

The objectives set out in this work cover two aspects. On the one hand, it would be interesting to be able to answer, in a well-founded manner, the following questions raised by crime in the city of Barcelona in recent times:

Q1 Is crime a seasonal phenomenon that depends on the month of the year? If so, in which months are crime rates highest and lowest?
Q2 Does this depend on the municipal district? (do different geographical units show different seasonal behaviour?). In other words: is the relationship between the month and the level of crime the same in all geographical units or are differences observed?
Q3 Can *seasonality* help predict crime? And if so, is it possible to build a predictive model that can be useful in this regard?

Second, and no less importantly, this paper aims to present the methodology used to answer the above questions in the particular case study, since its usefulness is completely general and can be applied in any region (or city), unit of time and geographical unit within the region, and could be of benefit to other researchers.

Regarding the tools used to try to achieve these objectives, this research considers *Predictability* and its components, *Constancy* and *Contingency*, which are measures introduced in [9] based on the mathematics of Information Theory that can be applied to any phenomenon known, or suspected to be, periodic or cyclical in time, and allow addressing the issue of determining to what extent the chosen time to study *seasonality* (months, in this case) determine the output variable, which is the level of crime categorized as low, medium and high. This is how questions Q1 and Q2 are addressed.

With regard to Q3, this paper addresses the issue of building, validating and comparing different predictive models of supervised machine learning for the level of crime in each municipal district that has shown to have significant values of the *seasonality* measures. For this, the methodology of Bayesian classifiers is used, which are learned from a data set that includes the level of crime as output (target) variable, and the month as the main input variable. A Bayesian classifier is a classifier that assigns each instance to the most likely class or category, that is, the class with the highest *a posteriori* probability given the evidence of the input variables for the instance [12], following the MAP (Maximum A Posteriori) criterion. Among them, we chose Naive Bayes, for its predictive effectiveness, despite its simplicity, both reasons for its widespread use to model complex real-world situations in many different areas, such as biomedical data [13], medicine [14] or robotics [15], among others. In criminology, Naive Bayes has been used for crime prediction which involves finding the most likely offender for a particular crime incident when the incident history is provided with the incident-level crime data in [16], and also for crimes related to social media such as Cyber Stalking, Cyber Harassment, Cyber Hacking and Cyber Scam in [17], and has been combined with a Support Vector Machine to identify criminal language for automatic text classification in [18], just to mention some recent works.

To control for the fact that crimes can follow different patterns depending on the day of the week (homicides are known to occur more frequently on weekends, for example) and that the frequency of the days of the week in each month varies depending on the year, additional input variables are introduced. Following [1, 19], these variables are defined by the counts for each of the weekdays of the month.

Since some studies conclude that monthly temperature variations do not have much influence on seasonal patterns of both robberies and homicides, such as [1], the decision has been made here not to include monthly average temperatures in the predictive model as input variables. Although their results show some variation between crime types, the authors argue that the *seasonality* of crime cannot be explained solely by changes in temperature, so it must be influenced by other factors, such as social activities. In addition, a compelling reason for this is that all geographical units have a very similar temperature, since they are located in the small area corresponding to the city of Barcelona, and that the temperature is highly correlated with the month of the year.

### State-of-the-art: Study of seasonal fluctuations in crime

There are different approaches related to the analysis of criminal patterns that can be found in the literature. In the first place, the *geographical aspect*, the one on which the analysis of hot spots is based, which identifies the areas with the highest criminal concentration. Secondly, the *temporal aspect* can be considered, which is usually studied with Time Series analysis. Most analytical studies on the *seasonality* of crime are based on the methodology of Time Series decomposition into its components (trend, *seasonality* and random error), or on its main alternative: a seasonal ARIMA analysis. As an advantage of the first methodology over the second, the classical decomposition provides the coefficients for each month, which makes it possible to determine which have a higher crime rate and which have a lower incidence, while the ARIMA methodology does not. Conversely, a weakness of the classical decomposition is that it assumes that non-stationarity (the deviation of the observations from the mean) is due to deterministic trends that persist over time. This assumption generally cannot be made in practice, since time series tend to show stochastic non-stationarity. A different approximation to that of ARIMA is given in [20], where an ecologically motivated stochastic differential equation based on the Lotka-Volterra model for predator-prey relationships is introduced to reproduce seasonal variations in aggravated assault, break in and theft from vehicles, burglary, grand theft auto, rape, robbery, theft and vandalism, using a data set from the Houston and Los Angeles metropolitan areas.

Finally, it is possible to have a combined *space-temporal approach*, which studies the interplay between time, space and the event of interest (the crime, in this case). The authors of [21] analyzed crime in Leeds, UK, based on clusters to identify where crime is concentrated and when (day of week and time of day). Areas with the highest crime density, as well as the season of the year when crime is highest, are identified in [22], and ARIMA analysis is used to predict crime in each region. They concluded that the season of the year is actually a good predictor of crime. [23] carried out a study to predict the location of crimes based on different variables such as the size of the population or the poverty index, and taking into account whether it was day or night, and the season of the year. Using three different classification methods (Logistic Regression, Neural Network, and an Ensemble model) and comparing them, they concluded that the forecast could be improved by making it monthly. Another example is [24], where crime mobility in Washington is predicted using Negative Binomial regression, taking into account time of day and day of week to better understand criminal mobility through the subway.

The present study is framed within the latter, that is, the *space-temporal* perspective, since temporal patterns are studied based on the geographical units (municipal districts), because each of them could have different characteristics that influence their criminal evolution over time. Since their introduction in [9], *Predictability* and its components, *Constancy* and *Contingency*, have been applied not only to describe aspects of periodicity in biological phenomena, but also to streamflow and rainfall data and hydrological time series [25]. In ecology [26] uses *Constancy* as a measure of flow stability, and [27] examines how the *Predictability* of temperature fluctuations explains the phenotype of planktonic marine microbes. In relation to adaptation to climate change, while [28] studies seasonal variations of the migratory strategy using *Contingency*, which is also used to relate the average weekly precipitation and daily maximum temperature, with the conflict and/or sociability in vertebrates derived from climatic uncertainty [29]. In [30] the influence of flow predictability on the thermal resilience of a river is studied.

In the field of social sciences, Colwell’s metrics have been used, for example, to define variables to which machine learning techniques can be applied to gain new insights into humanity’s transition to agriculture [31]. In particular, in Criminology few authors have studied the crimes based them. Specifically, the most extensive literature in this regard is found in Bogotá (Colombia), where temporal patterns in aggressive behaviours have been characterized [32, 33]. To the best of our knowledge, criminological studies such as this are novel and have not been conducted to date. Indeed, not only are these metrics used to study the *seasonality* of crime in the city of Barcelona, according to the municipal district, but they have also been combined with the use of supervised machine learning classification algorithms to predict the level of crime from the month of the year, in each district that shows significant value of Colwell’s metrics.

The organization of the rest of the manuscript is as follows. The “Methodological Approach” section first presents Colwell’s metrics *Predictability*, *Constancy* and *Contingency*, to evaluate *seasonality*. Second, it introduces Naive Bayes as a Bayesian network, which is a supervised machine learning classification algorithm, to predict the level of crime given the evidence of the month of the year and days of the week with 5 occurrences that month, at each geographical unit. Finally, it also presents the other state-of-the-art classifiers for comparison, the validation procedure, and the performance metrics. Section “The case study: Barcelona crime data” is devoted to presenting the data set with which the research has been carried out, while the “Results” section first presents some graphs of exploratory data analysis, then the results on the significance of Colwell’s metrics and, lastly, those related to the Bayesian classifier. The “Discussion” section delves into the implications and relevance of the results, and the body of the paper ends with a few words in the “Conclusions” section. S1 Appendix is devoted to the formal definition and properties of the Colwell’s metrics, based on Shannon’s entropy, and S2 Appendix to the compilation of some additional tables. After this, to finish, some notes from the authors, a list of abbreviations, the acknowledgements and funding sections, and references.

## Methodological approach

### *Predictability*, *constancy* and *contingency*: Assessing *seasonality*

This paper follows [9] to introduce three measures that can be applied to any phenomenon known or suspected to be periodic or cyclical in time and that can be scored in at least two states or levels. These measures are used to describe temporal patterns of the phenomenon, that is, to account for whether a seasonal pattern is repeated in all the years of the period considered.

*Predictability* *P*, which is the opposite of uncertainty, represents the relative certainty of knowing the level or state at a given time, and is defined as the sum of two separate components, which are *Constancy* *C* and *Contingency* *M*, so that *P* = *C* + *M*. *Constancy* *C* measures the certainty across tiers, that is, the degree to which a state stays the same throughout the season, and varies inversely with consistency across tiers. That is to say: more uniformity in the levels implies less *Constancy*. *Contingency* *M*, is the other additive component of *Predictability*, and represents the degree to which the unit of time unit (month) determines the level, that is, the degree of dependency on each other, and increases with the *mutual information* between them. In other words, *M* describes how closely the different levels correspond to the different months of the year and, therefore, it is the most informative measure of the degree of *seasonality* of the phenomenon under study. The maximum *Predictability* can be achieved as a consequence of either the maximum *Constancy* (the phenomenon is constant throughout the year), the maximum *Contingency* (the seasonal fluctuation is consistent throughout all the years), or a combination of them.

In the case study presented here, the phenomenon will be crime, specifically the level of crime against property in the city of Barcelona from 2010 to 2018, both included, which has been scored at *s* = 3 levels: low, medium and high, and is suspected to be cyclical on an annual basis. The data is displayed in the form of a frequency matrix or table, with the *t* = 12 months as columns, corresponding to the partition of a cycle (year) into time units, and *s* = 3 crime levels (states) as rows, for each geographical unit (municipal district).

Let (*m*_*ij*_)_*i*=1,…,*s*,*j*=1,…,*t*_ be the frequency matrix, with rows corresponding to levels, from low up to high, and one column per month, in the usual order from January to December. Here *m*_*ij*_ is the number of cycles (years, of the *w* = 9 years from 2010 to 2018) for which the number of crimes in the geographical unit was at level *i* in month *j*. As can be seen in Table 1, the usual dot notation for row and column sums is used:

$$m_{•j}=\sum_{i=1}^{s}m_{ij},\;m_{i•}=\sum_{j=1}^{t}m_{ij},\;N=\sum_{i=1}^{s}\sum_{j=1}^{t}m_{ij}=\sum_{j=1}^{t}m_{•j}=\sum_{i=1}^{s}m_{i•}$$

**Table 1:** General frequency matrix for *s* = 3 levels and the *t* = 12 months as a partition of the cycle (year). *m*_•*j*_ denotes the sum of the *j*th column, *m*_*i*•_ the sum of the *i*th row, and *N* the total sum.

	Jan	Feb	Mar	Apr	May	Jun	Jul	Aug	Sep	Oct	Nov	Dec	Total rows
low	*m*_11_	*m*_12_	*m*_13_	*m*_14_	*m*_15_	*m*_16_	*m*_17_	*m*_18_	*m*_19_	*m*_110_	*m*_111_	*m*_112_	*m*_1•_
medium	*m*_21_	*m*_22_	*m*_23_	*m*_24_	*m*_25_	*m*_26_	*m*_27_	*m*_28_	*m*_29_	*m*_210_	*m*_211_	*m*_212_	*m*_2•_
high	*m*_31_	*m*_32_	*m*_33_	*m*_34_	*m*_35_	*m*_36_	*m*_37_	*m*_38_	*m*_39_	*m*_310_	*m*_311_	*m*_312_	*m*_3•_
Total columns	*m*_•1_	*m*_•2_	*m*_•3_	*m*_•4_	*m*_•5_	*m*_•6_	*m*_•7_	*m*_•8_	*m*_•9_	*m*_•10_	*m*_•11_	*m*_•12_	*N*

Note that the column sums must all be equal to the number of years, which is *w* = 9, that is,*m*_•*j*_ = *w* for all *j* = 1, …, *t*, and therefore, *N* = *tw* (in this case, *N* = 12 × 9 = 108). The formal definition and some properties of the three measures are given in S1 Appendix. At this point the formulas to calculate them from the frequency matrix are given:

$$\begin{array}{c} Contingency\;\;M=\frac{1}{\;\text{log}\;s}\;(\;\;\text{log}\;t-\sum_{i=1}^{s}\frac{m_{i•}}{N}\;\;\text{log}\;\frac{m_{i•}}{N}+\sum_{i=1}^{s}\sum_{j=1}^{t}\frac{m_{ij}}{N}\;\;\text{log}\;\frac{m_{ij}}{N}\;)\;, \end{array}$$

$$\begin{array}{c} Constancy\;\;C=1+\frac{1}{\;\text{log}\;s}\;\sum_{i=1}^{s}\frac{m_{i•}}{N}\;\;\text{log}\;\frac{m_{i•}}{N}\;, \end{array}$$

$$\begin{array}{c} Predictability\;\;P=C+M=1+\frac{1}{\;\text{log}\;s}\;(\;\text{log}\;t+\sum_{i=1}^{s}\sum_{j=1}^{t}\frac{m_{ij}}{N}\;\;\text{log}\;\frac{m_{ij}}{N}\;)\;. \end{array}$$

To test the statistical significance of these measures, that is, whether *Predictability* is significant and to what extent *Constancy* and *Contingency* contribute to the predictability of the crime phenomenon, the corresponding statistical test of hypothesis are performed and p-values are calculated (see S1 Appendix for more details).

### Crime level prediction: A Bayesian classifier

The study of the *seasonality* of a phenomenon through the metric *Predictability* and its component *Contingency*, could lead to conclude, as in fact will be the case in this application, that the phenomenon is clearly seasonal in some of the geographical units, for which the next natural step is the construction of a predictive model from the time unit. In this case study, a predictive model is built to predict the level of crime from the month, for each municipal district that shows significant values of the metrics. Can a good model like that be built? What will be the predictive capacity of the unit of time (month) to infer the level of crime in a geographical unit (municipal district), without considering other characteristics, but simply a complementary temporal aspect such as the number of each of the days of the week in the month?

As explained in the Introduction, a supervised machine learning Bayesian classifier is built for any geographical unit (municipal district) that shows significant metrics, to predict the level of crime (low, medium or high) from the evidence given by only two input variables: the unit of time (month of the year), and the number of each of the days of the week in the month. As Bayesian classifier, the Naive Bayes (NB), which is a particular type of Bayesian network (BN), has been chosen.

BNs are graphical models that represent the probabilistic relationships between the variables that affect a phenomenon, and are used for probabilistic inference. For a set of random variables (discrete or categorical), a standard BN is a model that represents their joint probability distribution, the graphical part of the model consisting of a *directed acyclic graph* (DAG), whose nodes represent the random variables. The directed arcs between nodes represent conditional (not necessarily causal) dependencies governed by the **Markov condition**, which states that each node in the DAG is independent of those that are not its descendants, given its parents are known. *Bayesian Inference* is the term used to refer to the updating of probabilities of the network from a given piece of evidence: *a posteriori* probabilities are calculated from evidences and *a priori* probabilities. Prediction of a query variable given an evidence is the instantiation of the variable with the highest probability *a posteriori*, and this probability is said to be the *confidence level* of the prediction.

Naive Bayes is the simplest Bayesian Network since it has a fixed structure (DAG) that is not learned from the data, and assumes that the input variables are independent of each other given the class. This assumption can be unrealistic in many situations, although it has been seen in different studies that, despite this, it is a good classifier from the point of view of its predictive efficacy. To estimate the parameters (the conditional probabilities of any input variable conditioned on the class, and the marginal distribution of the latter), the Maximum Likelihood Estimation method is used.

Given the evidence of the month of the year and the days of the week with 5 occurrences that month (all other weekdays will have 4 occurrences, which is the minimum, 5 being the maximum), a Naive Bayes is learned from the data to predict the level of crime for a geographical unit, which is the level with the highest probability of those assigned by the model. Learning and prediction algorithms have been implemented in R language [34].

NB is compared with other state-of-the-art classifiers in the validation procedure: Neural Network (NN), Support Vector Machine (SVM) and Random Forest (RF), which have been built, respectively, with the mlNnet, mlSvm and mlRforest functions of the *mlearning* R software package [35], using the default values (max number of iterations = 1000 for NN, radial kernel for SVM, and 500 decision trees for RF). For the Two different options have been used for the NB but they give the same resulting model: the functions mlNaiveBayes from package *mlearning*, and naive.bayes from the R software package *bnlearn* [36], the first for its simplicity, and the second because this last package allows obtaining a measure of the strength of the arcs (equivalently, of the strength of the input variables) of the NB through its function arc.strength.

**Fig 1:**
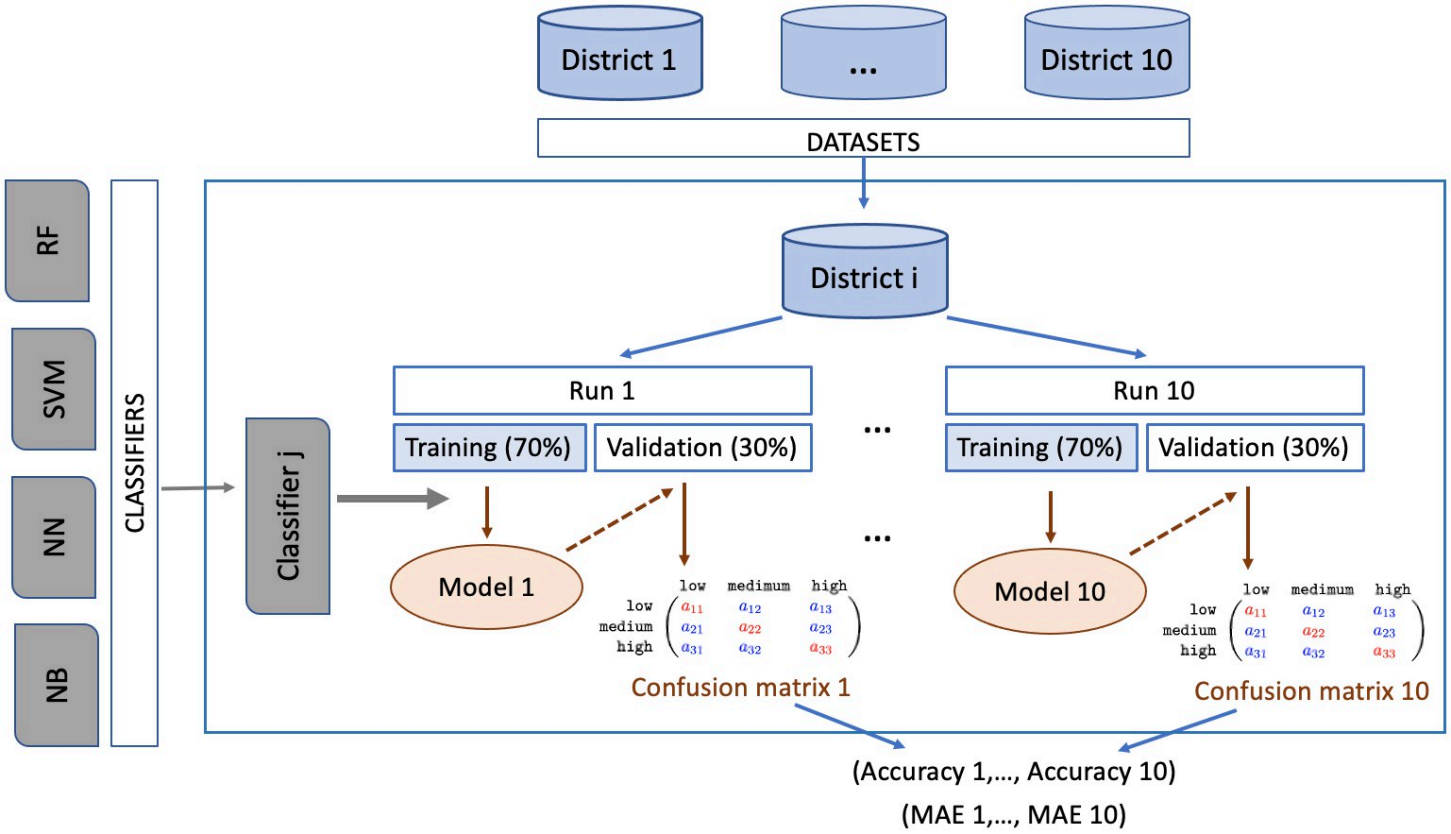
Construction, validation and comparison of predictive models: Implementation pipeline. For each geographical unit (municipal district) and classifier, a model is learned from the training data set for any run, and with the model and the validation data set, a confusion matrix is obtained, from which the two metrics (*accuracy* and MAE) are calculated.

It has been decided to perform a *split-validation* 70–30% procedure to compare the classifiers instead of the more popular *k*–fold cross-validation, since there are only 108 instances in the data set for any municipal district. The procedure of dividing the data set into training (70%) and validation (30%) data subsets is randomized. To reduce possible bias due to the (random) choice, the process is repeated 10 times (*runs*), using a different randomly selected seed in each case to perform the split on the data set. In this way, a confusion matrix is obtained for any model and run, and from it the following two performance measures are calculated, as shown in the pipeline in Fig 1.

- ***a*ccuracy** is considered the gold standard performance measure and is defined as the proportion of correctly classified instances in the validation set. The higher, the better.
- **Mean Absolute Error (MAE)** is the most popular metric for ordinal classification and is a measure of classification error. Therefore, the lower the better.

Formally, if a confusion matrix obtained in the validation procedure has the form

$$\begin{array}{cc} & \begin{array}{c} \text{observed}\\ \begin{array}{ccc} \text{low}\; & \text{medimum} & \;\text{high} \end{array} \end{array}\\ \text{predicted} & \left(\;\begin{array}{ccc} a_{11}\; & a_{12}\; & a_{13}\\ a_{21}\; & a_{12}\; & a_{23}\\ a_{31}\; & a_{32}\; & a_{33} \end{array}\;\right) \end{array}$$

where the rows correspond to the predicted levels, and the columns to the observed levels, the elements on the diagonal (*a*_11_, *a*_22_ and *a*_33_) are the number of correctly classified instances of each class, while off the diagonal the number of misclassified is recorded. Denoting by *V* the total number of cases in the validation set, $V=\sum_{i,j=1}^{3}a_{ij}\;,$ then,

$$accuracy=\frac{1}{V}\;\sum_{i=1}^{3}a_{ii}=\frac{1}{V}\;\left(a_{11}+a_{22}+a_{33}\right)\;,\;\text{MAE}=\frac{1}{V}\;\sum_{i,j=1}^{3}a_{ij}\;\left|i-j\right|\;.$$

Note that in the formula to obtain MAE, the elements of the main diagonal are irrelevant since *i* = *j*. Due to the term |*i* − *j*|, which is the weight of the error made when classifying an instance belonging to class *j* in class *i*, the error penalizes more the more the further the prediction is from the observed class, taking into account the ordering of the classes. For example, classifying an instance of class high as low, or vice versa, penalizes twice as much as classifying an instance of class medium as class low, or vice versa.

The four classifiers (NB, NN, SVM and RF) are built, one for each district, with the output variable (target) being the level of crime (low, medium and high), and input variables: Month of the year, and for each of the days of the week, from Sunday to Saturday, a binary variable indicating whether that day has 4 or 5 occurrences that month (which depends on the month and the year). Note that the year itself only enters the predictive model, indirectly, to generate these dummy binary variables.

## The case study: Barcelona crime data

**Fig 2:**
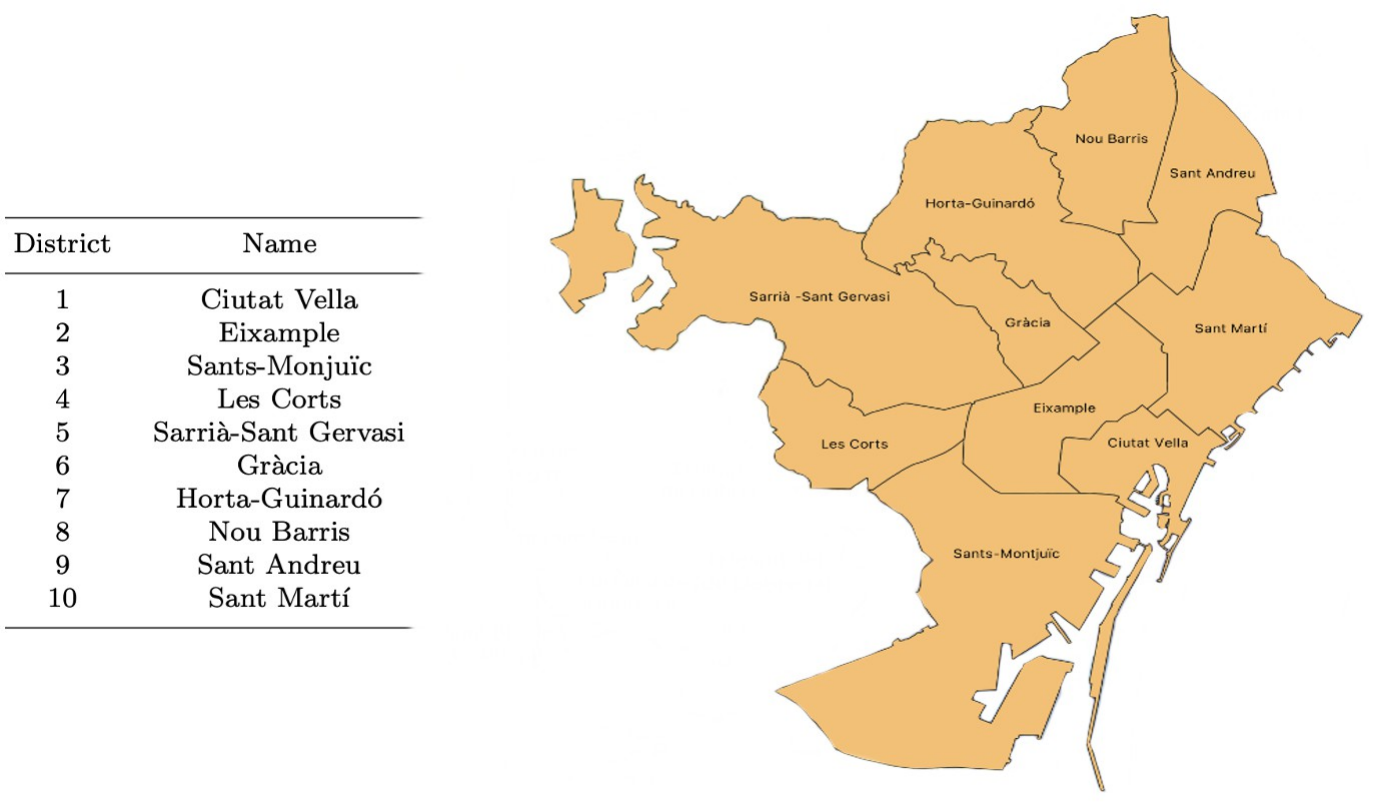
Map and names of the 10 municipal districts into which the city of Barcelona is divided, located on the Mediterranean coast of the Iberian peninsula.

To carry out an application of the methodology presented in this paper, the data set used contains information on different types of crimes in the city of Barcelona, located in the northeast of the Iberian Peninsula (see Fig 2), that covers the years between 2010 and 2018. The territorial division of the city of Barcelona is made up of 10 municipal districts whose names are recorded in Fig 2, which also shows the geographic location of the city and the districts in the city area.

All tables in this paper are of made bu the authors themselves, as well as all maps, which have been created using QGIS, which is an easy-to-use Geographic Information System (GIS), Open Code and licensed under GNU-GPL (http://www.qgis.org).

This paper explains an investigation at the “macro” level of geographic aggregation: the municipal districts. The reason is that the municipal districts are relatively homogeneous and their number is manageable if one tries to build a predictive model for each geographical unit, and study their differences regarding the seasonal behaviour of delinquency.

The macro-level approach seems to be appropriate in this case because in daily life, activities occur within a wider range of mobility than in a census section or street segment, and therefore criminal opportunities are greater in a district. Furthermore, the macro-level allows one to examine the interaction between units and how they are connected [37]. In fact, Cohen and Felson’s original Theory of Routine Activities was conceived at the macro-level [38], even though it was also considered at the micro-level. In addition, they obtained robust results for both levels of analysis when explaining crime [5]. In [39] authors also conducted a macro-level analysis of the relationship of routine activities to crime rates and concluded that “the results demonstrate quite convincingly that the leisure routine perspective can be profitably applied to explain aggregate crime rates”. Many other studies look at crime from a macro perspective, such as [40], whose research was based on 125 metropolitan areas in the United States, or [41], whose macro-level analysis of routine activities was successfully applied to arrests and searches by London police. At least in part, this revival of interest in macro-level approaches was encouraged by the intersection of four major scholarly contributions: the development of Cohen and Felson’s Theory of Routine Activities; the seminal work on inequality and violent crime [40]; the rediscovery in the 1980s of Shaw and McKay’s theory of social disorganization by scholars such as [42], and finally, the interest in deterrence theory at the macro-level (see for example [43]), which was a response to concerns about the impact of rapidly growing prison populations and motivated by parallel work by human capital economists.

The data set of information related to the total number of crimes has been structured by geographical unit, for each month from January 2010 to December 2018. That is, the basic units of time considered are months, covering a period of 9 full annual cycles (corresponding to 9 × 12 = 108 months), which will be seen to be sufficient to study seasonal and temporal patterns despite the random noise inevitably present in the data. Monthly data allow a deeper study of seasonal patterns than the typical quarterly aggregations (spring, summer, autumn, winter), or even bimonthly, which could mask *seasonality* due to offsets between the data of the different months that make up the aggregation.

**Fig 3:**
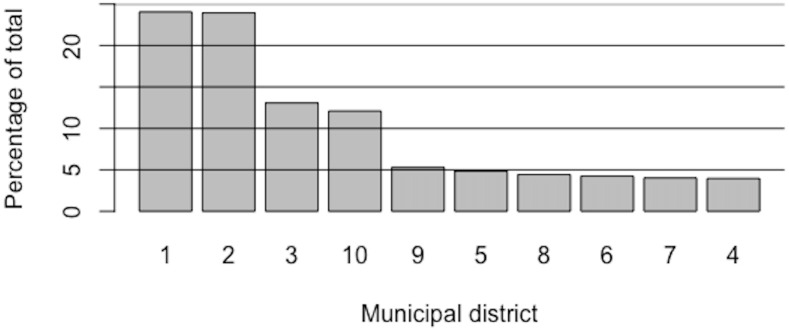
Crimes against property in Barcelona 2010–2018: Bar chart representation of the concentration by municipal district. Much of crime is concentrated in a relatively small fraction of districts (Pareto-like distribution).

The same could happen with multi-crime aggregations, which could also mask *seasonality*, so it seems convenient to work with disaggregated crime typologies, as long as they are not too few in the data set. In particular, the focus has been placed on crimes against property from a pragmatic thought based on logistics and resource management. In fact, it is the most relevant type of crime in terms of absolute frequency, and between 2010 and 2018, of the total of 1,756,810 crimes registered in Barcelona, 1,593,950 were crimes against property, which corresponds to 90.73% of the total. Furthermore, property crime has been chosen as the target because it is intuitively clear that it is likely to be affected by environmental factors, that is, to have a behaviour potentially dependent on geographic distribution, and will be shown to be so. In fact, as can be seen in Fig 3, more than 70% of the total crimes against property reported in Barcelona during this period were concentrated in only 4 out of 10 municipal districts. Specifically, districts 1, 2, 3, and 10 account for 73.17%, with the first two clearly standing out.

The following Table 2 shows the total number of crimes against property reported in the city of Barcelona from 2010 to 2018, by month and by year, according to police data.

**Table 2:** Number of crimes against property reported in Barcelona, from 2010 to 2018.

Year	Jan	Feb	Mar	Apr	May	Jun	Jul	Aug	Sep	Oct	Nov	Dec	Total
2010	13721	13030	14532	14218	15022	13937	15683	15759	14426	14656	13685	13473	172142
2011	12804	12561	13615	13564	15206	13960	15561	14423	13273	13655	13031	13139	164792
2012	12380	11709	12838	12985	14356	14219	15512	14133	13393	13557	12626	12544	160252
2013	12482	11429	12709	13001	13512	13382	14760	14265	13256	13131	12374	12608	156909
2014	12331	11841	12282	12446	13366	13379	14786	14105	13440	13237	11678	13088	155979
2015	12790	11343	12947	13378	14669	14288	14842	14151	13268	13421	12856	12910	160863
2016	12697	12775	12368	12473	13100	14350	15812	15368	14590	13438	13213	13237	163421
2017	12895	12210	13746	13206	15258	15052	17347	15409	14988	15668	16489	15971	178239
2018	16313	14928	16670	16543	17635	18523	19876	19254	18295	18719	18124	18258	213138
Total	118413	111826	121707	121814	132124	131090	144179	136867	128929	129482	124076	125228	1525735

### Introduction of crime level through categorization with z-scores

The number of crimes (against property) in each month of each of the years 2010–2018, in each district of the city, has been discretized to obtain a categorical output variable with three levels or classes: low, medium and high. The simplest approach would be to use the same cut-off points to categorize the data of all districts, but this is not as straightforward as it seems at first glance. The reason is that the orders of magnitude of the number or crimes are very different for the different geographical units (see Table 1 in S2 Appendix, where it can be clearly seen that there are four districts that stand out for their high incidence, 1, 2, 3 and 10, which are the ones located in the southeastern half of the city), and also for different years.

Then, to score the number of crimes in the three levels, the cut-off points have been standardized. To do this, first, the number of crimes corresponding to each municipal district and each year has been transformed into z-scores (subtracting the average and dividing by the deviation of the values for that district and year, so that the z-scores resulting are centered with unit deviation), then −0.675 and 0.675 are used as cut-off points. In this way, if the number of crimes followed an approximately normal distribution, the percentages of each of the levels would be, approximately, low: 25%, medium: 50%, high: 25%.

To improve the normality of the data, as long as there are no zeros in any of the monthly data, the natural logarithmic transformation (it is irrelevant what number is used as base of the logarithm) has previously been applied to the number of crimes. The logic that doing so effectively improves the normality of the data is supported by Table 2 in S2 Appendix, where the p-values of the Shapiro-Wilk normality test are found for the original data, as well as for the Ln-transformed data (data transformed by applying the natural logarithm to base *e*), for any municipal district, since in general the p-values increase by applying the logarithmic transformation.

Naturally, the choice of 25–50–25% is arbitrary, but it is fair and obeys an objective criterion, since it is applied equally to all geographical units and years, once the number of crimes has been transformed into z-scores, which greatly facilitates the procedure and makes it perfectly scalable. Of course, in this process of transforming the number of crimes into one of the three levels, information is lost, but interpretability and simplicity are gained. The values in the original data to which these cut-off points correspond have been recorded in Table 3 in S2 Appendix, and the following formula has been used for their calculation:

$$\begin{array}{cc} \text{Lower}\;\text{cut-off}\;\text{point:}\; & e^{\mu -0.675\;\sigma }\;,\;\text{Upper}\;\text{cut-off}\;\text{point:}\;e^{\mu +0.675\;\sigma } \end{array}$$

where *μ* and *σ* are the mean and standard deviation for each district and year, respectively, used to derive the z-scores.

## Results

**Fig 4:**
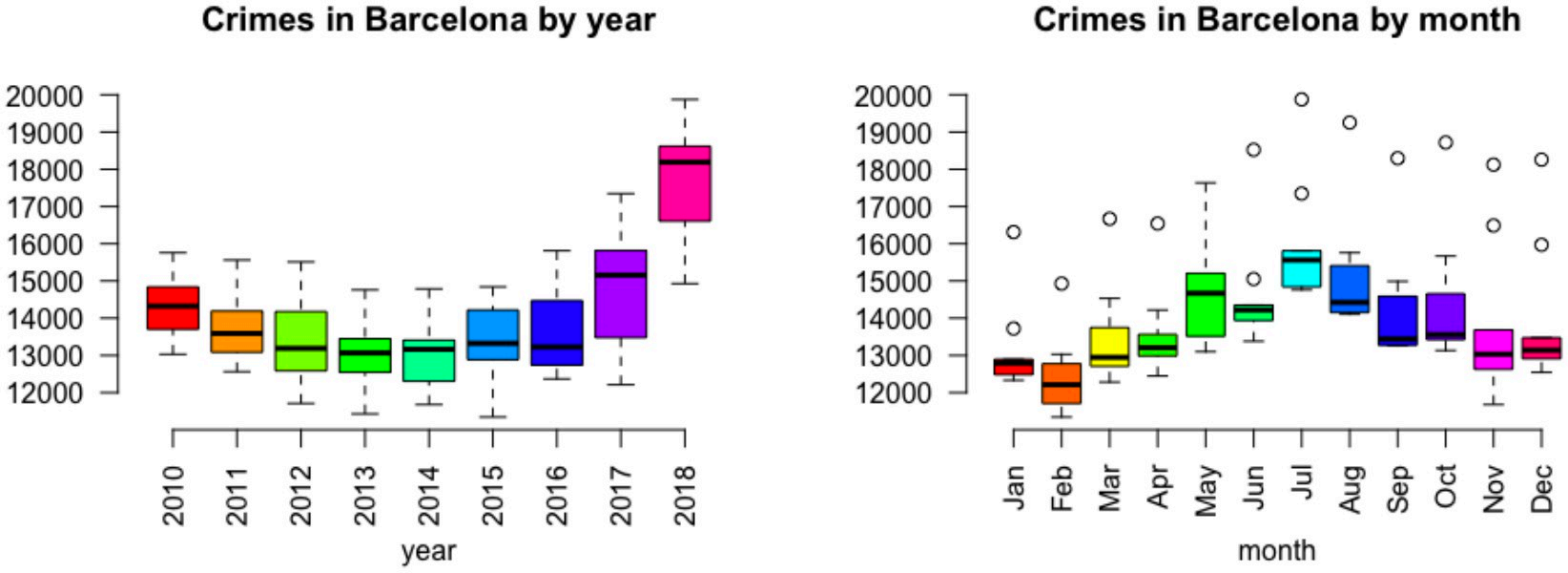
Boxplots of monthly crimes, Barcelona 2010–18, by year (left) and by month (right).

For the data in Table 2, the corresponding boxplots of the monthly occurrences for each year and of the annual events for each month are shown in Fig 4.

**Fig 5:**
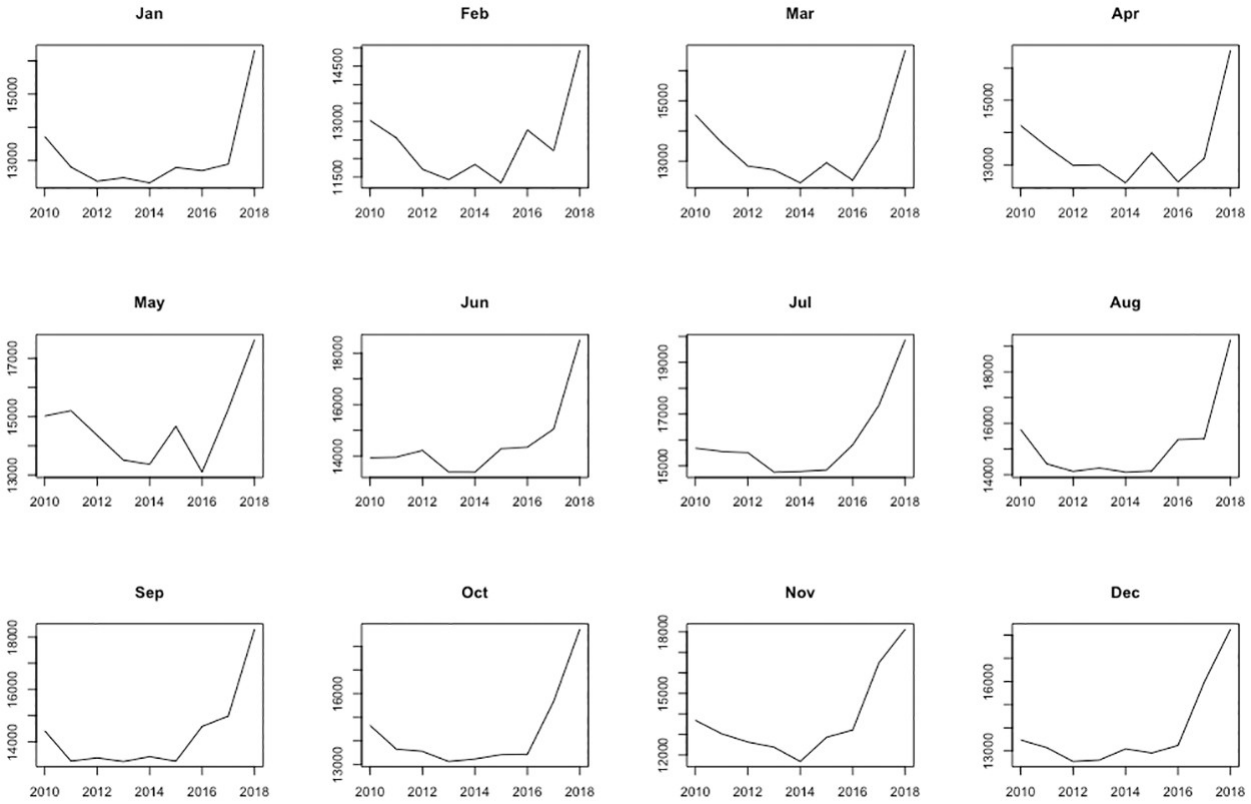
Temporary evolution 2010–18 of the monthly total of crimes in Barcelona, by month.

It is interesting to observe in Fig 4 that the occurrence of crime decreases from 2010 to 2014, and then rises again, shooting up quite a bit in 2018, indicating a temporal trend as the years go by. This phenomenon is not only observed at the year level, but also, although with slight differences, for each month, as can be seen in Fig 5.

It can also be observed the absence of outliers in the boxplots by year, that is, there are no anomalous months either due to too high or too low number of reported cases. By contrast, the boxplots by month show outliers, which in this case are years in which the number of crimes has been exceptionally high. This occurs in all months except May, which is clearly the month with the greatest variability. For the rest of the months, the year 2018 is always one of the atypical ones, the largest, the other being 2010 in January, and 2017 in June, July, November and December, in line with the increase in the number of crimes reported in recent years in the city.

**Fig 6:**
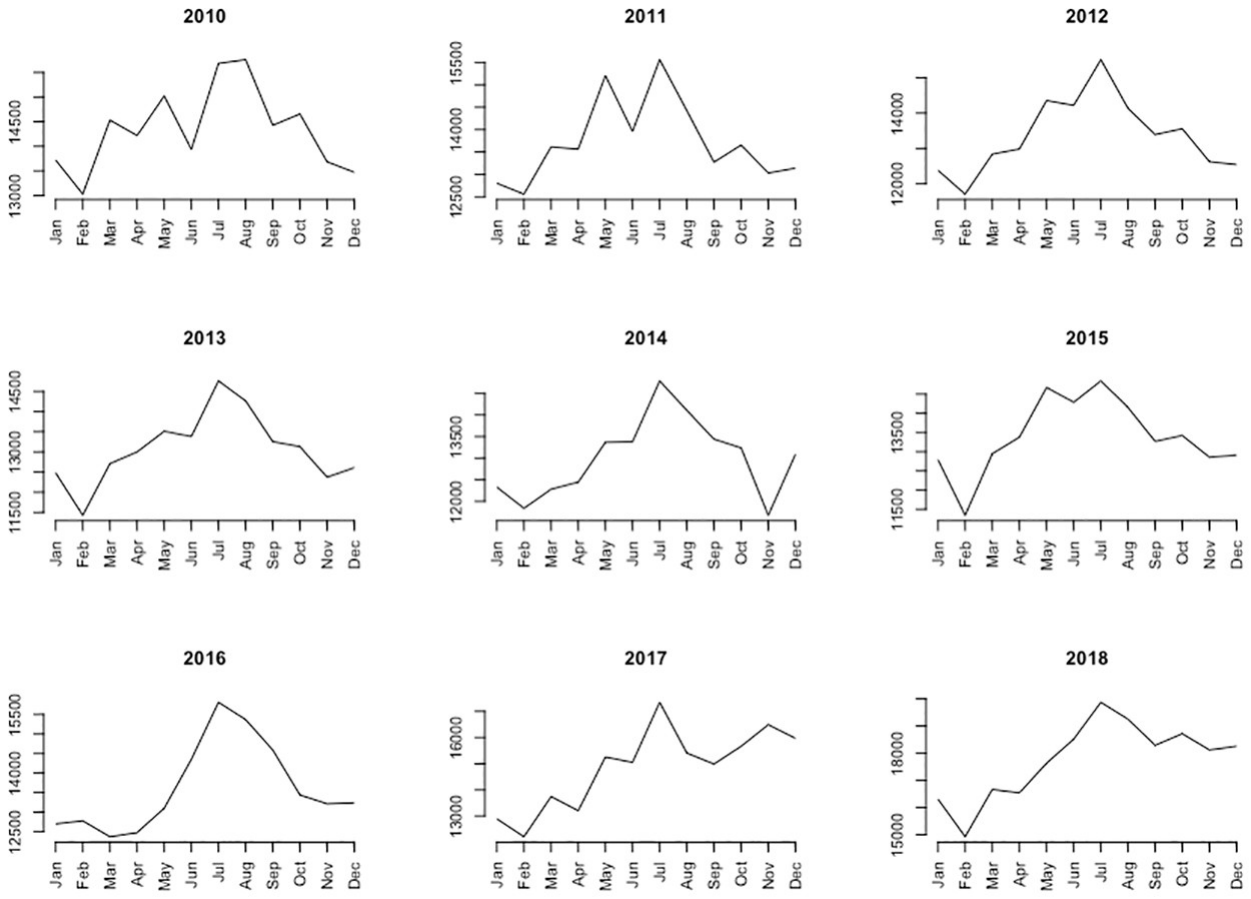
Seasonal trend from January to December of the monthly total of crimes in Barcelona, by year.

Fig 4 also shows a seasonal trend in the number of crimes, increasing until summer, to decrease again, which can also be observed every year (Fig 6). Some differences in the seasonal trend are observed throughout the period: in the early years (2010 to 2012), crimes increase in summer and decrease in winter. From 2013 to 2016, crime goes down after the summer, but not as much. In 2017 and 2018, crimes in the last months of the year have increased compared to previous years and reach almost summer levels.

**Fig 7:**
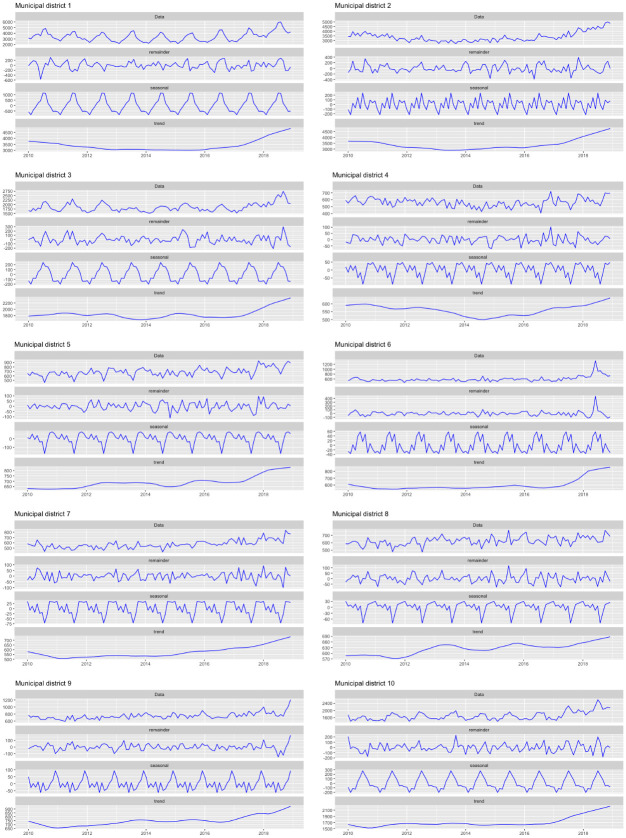
Additive decomposition of the time series of the number of crimes by district.

**Fig 8:**
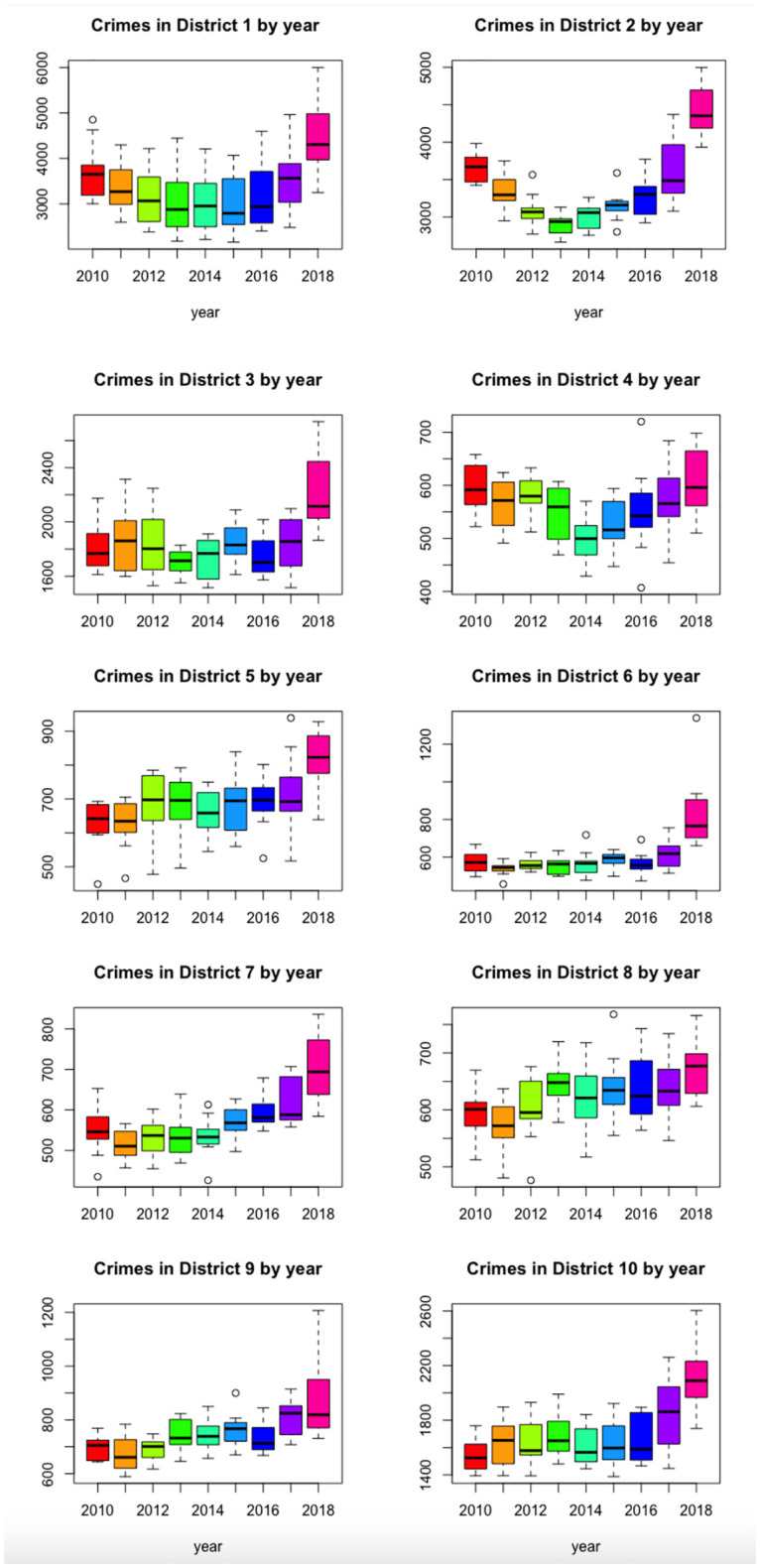
Boxplots of the monthly number of crimes in any municipal district, by year.

**Fig 9:**
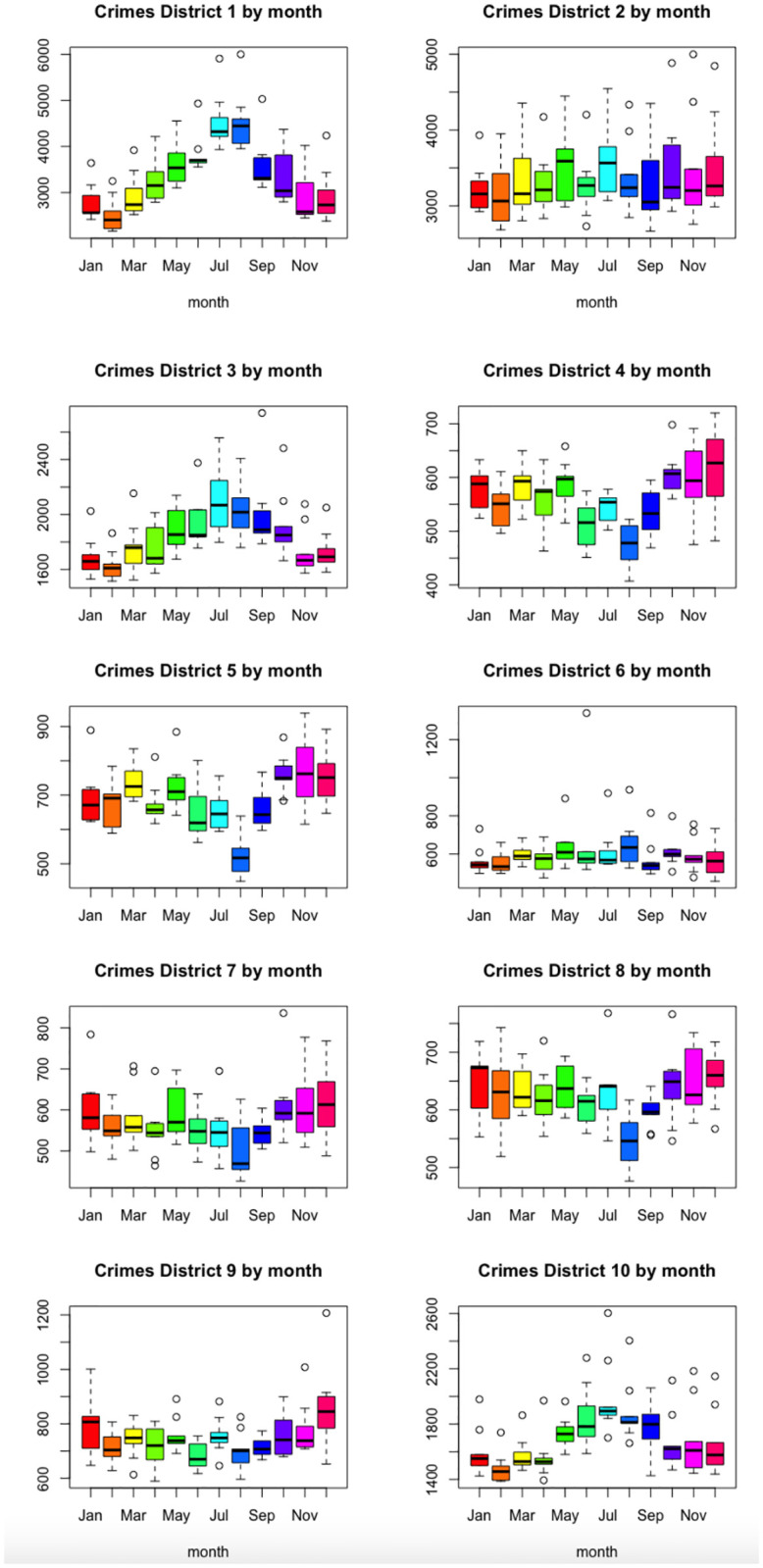
Boxplots of the monthly number of crimes in any municipal district, by month.

It is also possible to see the evolution of the number of crimes per year for the different municipal districts. Fig 7 shows, for each municipal district, the decomposition of the time series corresponding to the number of crimes, into seasonal, trend and irregular components (obtaining the decomposition by the function stl from the R software package *stats*). Fig 8 shows the boxplots of the total monthly crime in any district, by year, and in Fig 9 shows them by month. The behaviour of districts 1, 3 and 10 stands out, which clearly follow the same seasonal pattern as the city as a whole.

To finish this brief exploratory analysis, the asymptotic p-values for the statistical hypotheses tests with alternative hypothesis *H*_1_: correlation≠0, have been recorded, for the Pearson correlations between months (Table 3) and between years (Table 4), both corresponding to data in Table 2. Significant p-values are <0.05 (meaning *H*_1_ is accepted at a significance level of 5%).

**Table 3:** p-values for Pearson correlations between months.

	Feb	Mar	Apr	May	Jun	Jul	Aug	Sep	Oct	Nov	Dec
Jan	0.0005	0.0000	0.0000	0.0021	0.0004	0.0016	0.0000	0.0003	0.0003	0.0066	0.0033
Feb		0.0022	0.0042	0.0196	0.0047	0.0027	0.0000	0.0006	0.0030	0.0156	0.0095
Mar			0.0000	0.0000	0.0029	0.0023	0.0007	0.0029	0.0002	0.0038	0.0041
Apr				0.0002	0.0023	0.0063	0.0012	0.0048	0.0011	0.0131	0.0108
May					0.0030	0.0038	0.0097	0.0144	0.0010	0.0041	0.0055
Jun						0.0000	0.0003	0.0000	0.0000	0.0008	0.0003
Jul							0.0002	0.0000	0.0000	0.0000	0.0000
Aug								0.0000	0.0001	0.0019	0.0008
Sep									0.0000	0.0007	0.0001
Oct										0.0000	0.0000
Nov											0.0000

**Table 4:** p-values for Pearson correlations between years. In bold are p-values ≥0.05 (no statistically significant correlation).

	2011	2012	2013	2014	2015	2016	2017	2018
2010	0.0004	0.0006	0.0000	0.0019	0.0017	0.0208	**0.0950**	0.0165
2011		0.0000	0.0002	0.0018	0.0000	0.0425	**0.0513**	0.0274
2012			0.0000	0.0001	0.0000	0.0038	0.0185	0.0023
2013				0.0000	0.0000	0.0016	0.0247	0.0009
2014					0.0014	0.0003	0.0339	0.0017
2015						0.0303	0.0271	0.0040
2016							0.0225	0.0013
2017								0.0000

From the p-values in Table 3 it can be seen that the correlation coefficients for all possible pairs of months (columns in Table 2) are significantly different from zero (in fact, they are positive), which means that the behaviour over the years for the different months is similar. For example, if from the year 2010 to 2011 there is a decrease in the number of crimes recorded in January, there will likely also be a decrease in the other moths (in this example, this turns out to be true for all months except May and June).

All p-values in Table 4, corresponding to the Pearson correlations for all possible pairs of years, are < 0.05 (which means that the correlation coefficients are significantly different from zero, positive, in fact) except for two: between 2010 and 2017, on the one hand, and between 2011 and 2017 on the other. That is, the behaviour throughout the months for the different years is similar except for the year 2017, which is not, compared to 2010 or 2011, but neither does it have a strongly resembling behaviour to that of the rest of the years (p-values between 0.01 and 0.04, that is, significant but not much), except for the year 2018, which has a significantly positive correlation with 2017 (according to what can be observed in Fig 6).

### *Predictability*, *constancy* and *contingency*: Assessing *seasonality*

Crime in Barcelona may well be periodic or cyclical over time. In fact, Fig 6 shows a cyclical character since for any year between 2010 and 2018, crime increases in spring-summer, and decreases again in autumn (although in the years 2017 and 2018, there is a rebound in crime in the last months of the year). The study of *Predictability* and its components *Constancy* and *Contingency*, as measures related to temporal patterns, could be carried out at any geographical level: for the entire city, by municipal districts, neighborhoods or census sections, although in this work the focus is on municipal districts. It have already been explained how crime data has been scored at low, medium or high levels before organizing it into a frequency table for any geographical unit, with months as columns and crime levels, which are states, as rows, as shown in Table 5.

**Table 5:** Frequency matrix by municipal districts. For any district and month, the number of the 9 years of the considered period (2010–18) that are in each of the three levels corresponding to the rows is recorded.

		Jan	Feb	Mar	Apr	May	Jun	Jul	Aug	Sep	Oct	Nov	Dec	Total rows
Dist. 1	low	9	9	5	0	0	0	0	0	0	0	6	5	34
	med.	0	0	4	9	7	4	0	0	7	9	3	4	47
	high	0	0	0	0	2	5	9	9	2	0	0	0	27
Dist. 2	low	4	8	2	4	1	3	0	0	4	0	3	1	30
	med.	5	1	5	5	5	6	3	7	4	7	4	4	56
	high	0	0	2	0	3	0	6	2	1	2	2	4	22
Dist. 3	low	6	8	4	5	1	0	0	0	0	0	3	5	32
	med.	3	1	5	3	6	4	1	3	2	7	6	4	45
	high	0	0	0	1	2	5	8	6	7	2	0	0	31
Dist.4	low	0	2	0	2	0	7	1	9	3	0	0	0	24
	med.	5	6	5	5	5	2	8	0	6	3	4	3	52
	high	4	1	4	2	4	0	0	0	0	6	5	6	32
Dist. 5	low	1	1	0	1	0	4	3	9	1	0	1	0	21
	med.	6	7	5	8	6	5	6	0	8	2	0	2	55
	high	2	1	4	0	3	0	0	0	0	7	8	7	32
Dist. 6	low	4	5	1	5	0	1	0	1	5	1	2	3	28
	med.	4	3	5	2	6	7	9	3	4	4	6	5	58
	high	1	1	3	2	3	1	0	5	0	4	1	1	22
Dist. 7	low	0	3	0	4	0	5	2	8	2	0	0	1	25
	med.	5	5	8	5	6	3	7	1	7	3	2	2	54
	high	4	1	1	0	3	1	0	0	0	6	7	6	29
Dist. 8	low	1	2	0	3	1	1	2	8	4	1	0	0	23
	med.	4	5	7	3	5	8	6	1	5	5	6	7	62
	high	4	2	2	3	3	0	1	0	0	3	3	2	23
Dist. 9	low	0	4	1	5	1	6	2	4	4	1	0	1	29
	med.	4	4	7	3	5	3	3	5	5	7	5	1	52
	high	5	1	1	1	3	0	4	0	0	1	4	7	27
Dist. 10	low	4	8	3	6	0	0	0	0	1	2	3	3	30
	med.	4	1	6	3	8	4	0	0	4	7	5	6	48
	high	1	0	0	0	1	5	9	9	4	0	1	0	30

**Fig 10:**
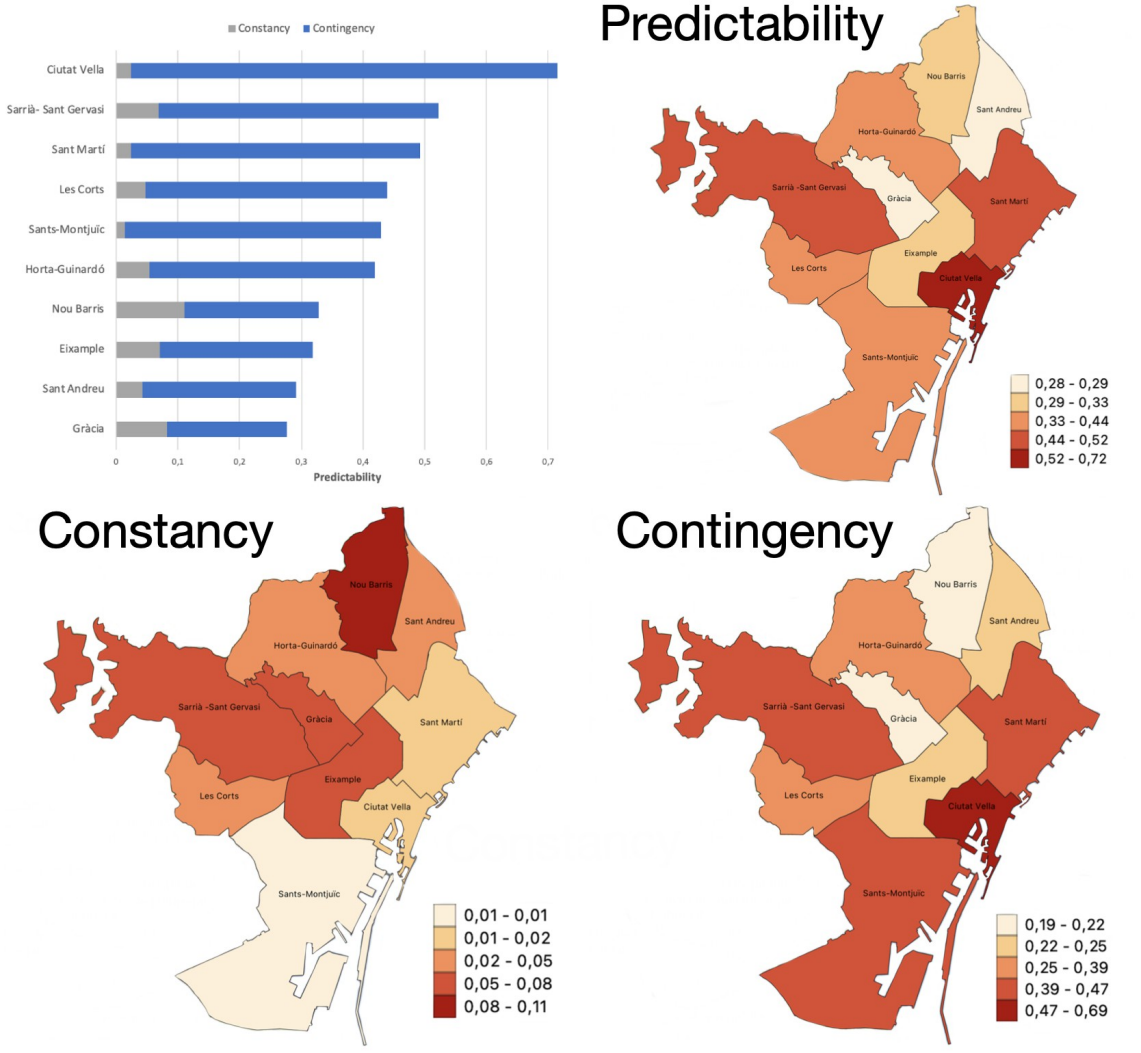
Top left: Bar chart of the distribution of *Predictability* and its components by districts (Table 6). Color map of *Predictability* (top right), *Constancy* (bottom left) and *Contingency* (bottom right).

From data in Table 5, the values of the three measures are calculated: *Predictability* *P*, *Constancy* *C* and *Contingency* *M*, as well as the corresponding p-values (see S1 Appendix), which are recorded in Table 6. It can be seen that all but district 3 show a statistically significant *Constancy* (significantly >0), and all districts show a highly statistically significant value of *Contingency* and *Predictability*, which means that using the month of the year as input helps to predict the level of crime, in any municipal district. The “*C*/*P*” column indicates the percentage of *Predictability* that *Constancy* represents, and similarly with *Contingency* in the “*M*/*P*” column. The “Rank” column gives the rank order of the 10 municipal districts according to the value of *Contingency* *M*, from the highest (1) to the lowest (10). See Fig 10 for a graphical representation of these measures, including color maps.

**Table 6:** *Predictability* *P*, *Constancy* *C* and *Contingency* *M*, and p-values for the statistical test with alternative hypothesis *H*_1_: *The measure is* >0, by municipal districts (data in Table 5). In bold the only non-significant p-value. Unless otherwise stated, p-values are significant at 0.1% (***).

Dist.	*P*	p-value	*C*	p-value	*M*	p-value	*C*/*P*	*M*/*P*	Rank
1	0.71503	0.00000	0.02377	0.01709*	0.69126	0.00000	3.325%	96.675%	1
2	0.31900	7.7482×10^−13^	0.07112	5.16199×10^−6^	0.24788	2.52694×10^−9^	22.295%	77.705%	8
3	0.43011	0.00000	0.01380	**0.09427**	0.41631	0.00000	3.208%	96.792%	4
4	0.44023	0.00000	0.04738	3.00500×10^−4^	0.39285	0.00000	10.762%	89.238%	5
5	0.52324	0.00000	0.06929	7.05658×10^−6^	0.45395	0.00000	13.243%	86.757%	3
6	0.27724	2.18326×10^−10^	0.08251	7.34239×10^−7^	0.19473	2.19113×10^−6^	29.763%	70.237%	10
7	0.41925	0.00000	0.05485	8.35656×10^−5^	0.36440	2.22045×10^−16^	13.084%	86.916%	6
8	0.32782	2.29594×10^−13^	0.11037	6.24103×10^−9^	0.21745	1.29830×10^−7^	33.667%	66.333%	9
9	0.29190	3.08644×10^−11^	0.04285	6.52711×10^−4^	0.24905	2.16425×10^−9^	14.679%	85.321%	7
10	0.49249	0.00000	0.02418	0.015927*	0.46831	0.00000	4.911%	95.089%	2

### Crime level prediction: A Bayesian classifier

A *split-validation* 70–30% procedure has been performed to compare the four classifiers (NB, RF, SVM and NN) built for any of the 10 municipal districts, and 10 runs of this procedure have been performed using different seeds to randomly split the data set into disjoint training and validation sets. Next, for each municipal district, 10 confusion matrices have been obtained, one for each run, and from them both *accuracy* and MAE have been calculated. The averages are recorded in Table 7.

**Table 7:** Mean values of *accuracy* and MAE, by classifier and municipal district. Ranking of the four classifiers in brackets from 1 (best) to 4 (worst), and number of districts in which each classifier ranks first.

District	NB		RF		SVM		NN	
	*accuracy*	MAE	*accuracy*	MAE	*accuracy*	MAE	*accuracy*	MAE
1	0.77489 (**1**)	0.18947 (**1**)	0.69503 (2)	0.25789 (2)	0.53113 (4)	0.41579 (4)	0.56912 (3)	0.37105 (3)
2	0.57036 (2)	0.36842 (2)	0.56603 (3)	0.37368 (3)	0.62150 (**1**)	0.34211 (**1**)	0.49973 (4)	0.47895 (4)
3	0.52385 (**1**)	0.40789 (**1**)	0.50096 (2)	0.44737 (2)	0.46791 (3)	0.50526 (3)	0.39844 (4)	0.56579 (4)
4	0.53494 (**1**)	0.38947 (**1**)	0.49795 (2)	0.42632 (2)	0.42807 (3)	0.52368 (4)	0.39617 (4)	0.52105 (3)
5	0.68201 (**1**)	0.27632 (**1**)	0.60408 (2)	0.35000 (2)	0.54631 (4)	0.42105 (4)	0.57986 (3)	0.41316 (3)
6	0.44350 (4)	0.55263 (4)	0.51027 (**1**)	0.45526 (**1**)	0.44944 (3)	0.48684 (3)	0.46334 (2)	0.47105 (2)
7	0.60050 (**1**)	0.36842 (2)	0.56923 (2)	0.36579 (**1**)	0.51801 (3)	0.43421 (3)	0.48841 (4)	0.46316 (4)
8	0.56973 (**1**)	0.39211 (4)	0.55205 (2)	0.37632 (**1**)	0.53116 (4)	0.38947 (3)	0.54543 (3)	0.38158 (2)
9	0.50232 (**1**)	0.46316 (**1**)	0.46248 (2)	0.48684 (2)	0.45112 (3)	0.49211 (3)	0.39134 (4)	0.55263 (4)
10	0.61662 (2)	0.33158 (2)	0.62461 (**1**)	0.32368 (**1**)	0.41883 (4)	0.52632 (4)	0.44293 (3)	0.51842 (3)
ranks 1st	7	5	2	4	1	1	0	0

The numbers in brackets in Table 7 indicate the ranking of the four classifiers, for each municipal district and performance metric, where **(1)** corresponds to the best, and (4) to the worst, and the last row of the table shows the number of districts, out of the 10 possible, for which the classifier occupies the first position in the ranking, for each of the two metrics.

To make the comparison between the four types of classifiers, a F test (ANOVA) has been carried out, and it has not been significant, that is, no statistically significant differences are detected between the four at the same time, for any district and neither of the two metrics. Taking a look at the ranking, it is evident that the two classifiers that stand out positively are NB and RF, in that order. Are the differences between them significant? And if so, in what sense, that is, which is preferable, from a predictive point of view?

To answer these questions, a paired t-test can be performed to compare the means, after a previous Shapiro-Wilk normality test that does not allow to rejecting normality. One-sided p-values for the pairwise comparison between NB and RF, for each district and metric, were recorded in Table 8.

**Table 8:** One-sided p-values for NB versus RF, with paired t-test. In the column “NB>RF” for the alternative hypothesis that the mean of the metric is greater for NB than for RF. And vice versa in the “NB<RF” column. Significant p-values (<0.05 or slightly above) are in bold. For *accuracy*, the higher the mean, the better, while for MAE, the opposite is true.

District	*accuracy*		MAE	
	NB>RF	NB<RF	NB>RF	NB<RF
1	**0.01222777**	0.9877722	0.9877722	**0.01222777**
2	0.41145010	0.5885499	0.5934534	0.40654665
3	0.25979288	0.7402071	0.8219469	0.17805314
4	0.10348069	0.8965193	0.9185585	0.08144154
5	**0.00096577**	0.9990342	0.9994593	**0.00054073**
6	0.99253495	**0.0074651**	**0.0010567**	0.99894335
7	0.16443800	0.8355620	0.4603108	0.53968922
8	0.30263383	0.6973662	0.4090538	0.59094623
9	**0.05759570**	0.9424043	0.8146889	0.18531113
10	0.60734917	0.3926508	0.3899989	0.61000114

In Table 8 it can be seen that for *accuracy*, NB is significantly better than RF for districts 1, 5 and 9, and worse for District 6. With regarding MAE, NB is significantly better for districts 1 and 5, slightly better for District 4, and worse for District 6. Overall, an advantage is observed for NB, since RF is the better for only one district, while NB is better for three, without statistically significant differences for the rest. This leads to the choice of NB, which also has the advantage of being simpler, as a predictive model to learn from the entire data set for future predictions.

Therefore, a different NB classifier is learned from the entire data set for each district. With the function arc.strength of the R software package *bnlearn*, the strength of the directed arcs (equivalently, of the input variables) of the NB model has been obtained, measured by the gain/loss of score which would be caused by arc removal, with multinomial log-likelihood (loglik) scoring, which is equivalent to using the Akaike Information Criterion or the Bayesian Information Criterion scoring in our case. In other words, the strength of the directed arc is the difference between the scores of the network in which the arc is and is not present, and then, the stronger the more negative. The strength of the input variable “month” is recorded in Table 9 below for any municipal district, and also the corresponding p-values, which are all significant (<0.05). In contrast, the p-values for the strength of the binary inputs for the days of the week are not significant (i.e., they are greater than 0.05), with a few exceptions, so they are not reported.

**Table 9:** Arc strength of the input variable “month” in the NB model, for any municipal district, and the corresponding p-values.

District	loglik gain/loss	p-value	Ranking position
1	−82.01808	1.035118 × 10^−23^	1
2	−29.41058	3.333507 × 10^−05^	8
3	−49.39551	1.048398 × 10^−11^	4
4	−46.61190	9.632986 × 10^−11^	5
5	−53.86077	2.809240 × 10^−13^	3
6	−23.10416	1.859022 × 10^−03^	10
7	−43.23579	1.355811 × 10^−09^	6
8	−25.80056	3.547828 × 10^−04^	9
9	−29.54976	3.034139 × 10^−05^	7
10	−55.56448	6.935020 × 10^−14^	2

Compare Table 9 with Table 6 to see that the district ranks for the strength of *Contingency* and that of “month” as a predictor in the NB model, they match perfectly. The probability of this happening simply by chance is extremely low, 1 in 10! = 3,628,800. Put in context, this probability is much lower than, for example, drawing a particular combination of 5 cards in a poker hand, for example a *royal flush* of hearts, since the number of different possible hands is (525)=2,598,960.

In addition, a surprising result should be highlighted: the dependence between the strength of the “month” as a predictor of the level of crime, measured with the loglik gain/loss of the NB model, changed sign (Table 9), and the value of *Contingency* *M* (Table 6) is almost perfectly linear. In fact, the correlation is 1, and the regression line that makes it possible to predict from the *Contingency* the predictive strength of the “month” for the level of crime in a given municipal district, is:

$$-\;\text{loglik}\;\text{gain/loss}\;=4.493867\times 10^{-14}+0.01186501\times M$$

As an illustration of the application of the built predictive model (NB), it is possible to fix a district and a month, and without entering any value for the binary variables of the days of the week, the prediction of the Odds in favor of having a high level of crime can be obtained. Table 10 shows the predicted Odds Ratio (OR) with respect to January in favor of the high crime level, for any of the months, both for District 1 and District 7, chosen as examples because they exhibit a very different behaviour, obtained from their respective NB classifiers.

**Table 10:** Predicted OR from January in favor of the high crime level for any month, for districts 1 and 7. OR > 1: Months with Odds greater than January’s. OR < 1: months with Odds less than January’s. OR = 1: months with Odds equal to January.

Month	District 1	District 7
January	1	1
February	1	0.16
March	1.01	0.16
April	1	0
May	95.27	0.63
June	415.31	0.16
July	37230.45	0
August	37230.45	0
September	95.27	0
October	1	2.49
November	1.01	4.34
December	1.01	2.51

## Discussion

The methodology used in the research and exposed in this paper allows the study of phenomena such as crime, susceptible to presenting, as actually happens, *seasonality* (cyclical patterns that repeat at regular intervals). Although it has been carried out over a period of 9 years of monthly data, corresponding to crimes against property committed in the municipal districts of the city of Barcelona, a strength of the study is that it can be extrapolated to the consideration of any type of crime in any historical-geographical context. The strategy used to score the characteristic of interest (the number of crimes) into labels or categories also seems to be of general and intrinsic interest. The two aspects that make up this methodology are:

1. Characterization of crime *seasonality* using Colwell’s *Contingency* *M* as a measure of the predictability of the crime level from the month.*M* turns out to be a useful measure of *seasonality* since high values indicate strong differences between crime levels throughout the months of the year, and its values are significantly positive in all municipal districts, ranging from 0.19473 for District 6 (70.273% of the *Predictability*) and 0.69126 for District 1 (96.675% of the *Predictability*).
  It is of interest in itself that this measure of the degree to which a phenomenon is linked to specific seasons, can be exported from biological or physical events such as flowering or rainfall, where it is usually applied, to the consideration of complex human behaviours that are the object of study of the social sciences, such as crime.
2. Construction and comparison of different supervised machine learning classification algorithms (Naive Bayes, Neural Network, Support Vector Machine, Random Forest) in a validation procedure, to choose the one with the best predictive performance for the crime level, from the month of the year and the number of weekdays in the month, which happens to be the Naive Bayes.

The methodology introduced has given the opportunity to address the issues raised in the objectives of the study. Specifically, on one hand, it has been possible to observe that crimes exhibit a temporal pattern of *seasonality* according to the month of the year, that is, it has been possible to answer affirmatively to question Q1. Besides, it was found that considering Barcelona as a whole, crime reaches its peak every year in July and it falls to its minimum in February (except in 2016, when it occurred in March). The years 2017 and 2018 show local peaks in the October-December period, in line with other studies such as [44]. From an interpretative point of view, it could be ventured that this phenomenon is due, at least in part, to Christmas holidays and other shopping events such as the *Black Friday*. In fact, summer vacations and Christmas dates could contribute significantly to the *seasonality* of crime, among other activities that could help understand criminal behaviour, but these speculations are outside the scope of the present study.

On the other hand, in this paper *seasonality* has been studied from the spatial perspective at the macro level, based on the municipal districts, which are the largest geographical unit of analysis of the city of Barcelona. In Ecological Criminology, the accepted premise is that the smaller the unit of analysis, the better, since using a larger unit such as the district translates into greater variation within it with respect to what it would have meant using smaller units, such as neighbourhood or census section. Although for this reason the choice of the geographical unit at the macro level could be seen as a weakness, the consideration of the cost-benefit balance according to the computational effort and the difficulty in interpreting the results at the micro level have advised the approach that we have carried out. Furthermore, this limitation does not pose a serious threat since very promising results have been obtained.

In fact, it has been found that there are differences in seasonal fluctuations according to the municipal district, some of them having much more extreme oscillations than others, and having a clearly different seasonal behaviour between them, in response to question Q2. If the districts are grouped into: **maritime** (districts 1, 3 and 10), **internal** (districts 2 and 6), and **external** (districts 4, 5, 7, 8 and 9) (see Fig 2), it turns out that property crimes in **maritime** districts peak in summer and fall in winter, while **internal** districts peak in summer but also in spring and autumn, and **external** districts have peaks in autumn-winter. In other words, **maritime** districts behave like the entire city, and for them it can be concluded that crimes against property occur more frequently in summer.

These results are consistent with [8], where the authors found that property crime was more likely to occur in Minneapolis during the summer, when temperatures are higher, citizens are on vacation, and schools are closed. In [45] it is also explained that contemporary literature has observed higher rates of property crime during the summer. Specifically, property crimes were more likely to occur in parks in the summer and spring, while in winter they were more likely to occur at drinking establishments, which have limited capacity and surveillance cameras. Then, since physical interaction with strangers is less likely, property crime tends to be lower in winter. On the other hand, in summer people tend to congregate more and spend more time away from home. This is in line with the Theory of Routine Activities, since crime is concentrated in the most touristic places in Barcelona and in summer is when more opportunities appear: the more crowded places and the more people who do not know each other, the less surveillance and the greater abundance of desired objects, which create the opportunity to commit a crime when the offender is motivated.

Finally, in all the districts the *Contingency* *M* has turned out to be statistically significant and the input variable “month”, a good predictor of the crime level with the Naive Bayes, evaluating the performance of the classifier through both the *accuracy*, which is the gold-standard metric, and the *Mean Absolute Error* (MAE), which is a metric designed for ordinal-type class variables, such as our case, since the level of crimes has been scored at low/medium/high. So, the answer to question Q3 is yes, that is, *seasonality* helps predict the level of crime, and a predictive model can be built from the input variables “month” and the binaries of the days of the week.

## Conclusions

Crime is a complex social problem throughout the world, which causes insecurity and fear of crime. In particular, the occurrence of crimes against property in the urban space generates social alarm and a perception of insecurity among citizens that translates into changes in the patterns of social behaviour and can affect tourism and commerce in cities.

This paper focuses on the introduction of a new methodology for the study of *seasonality* and its application to the crime data set of Barcelona in the period 2010–2018, with geographical units at the “macro” level (municipal districts), to build a predictive model of the level of crime. This methodology combines the simplicity of entropy-based Colwell’s metrics and the power of machine learning. Presenting this new methodology is an objective of the work, since it is of general application in the context of the study of a phenomenon that evolves over time and is likely to have a seasonal or cyclical behaviour, of which it is interesting to study a qualitative characteristic (or that can be scored in states).

The results of the study have been positive, but this has not been surprising. What is really surprising, given that Colwell’s metrics and Naive Bayes are technically very different, is that they have turned out to be intrinsically linked in terms of results. That is why entropy-based metrics and supervised machine learning classification algorithms, which are the tools that this methodology combines, can be considered as “two sides of the same coin”.

There are different aspects related to this research that we would like to continue working on in the future, such as using more historical data to corroborate the results obtained and make them more robust, or detecting different patterns over the years. It would also be interesting to compare the behavior of the city of Barcelona with other European or Mediterranean cities to see if they share criminal patterns or, on the contrary, Barcelona shows some singularity.

In conclusion, the methodology presented in this paper has proven to be suitable for investigating *seasonality* and temporal patterns, being useful for a better understanding of criminal dynamics in the city and for generating local predictive models, capable of adapting to time and place, which can help authorities prevent crime, manage police resources, plan police interventions and improve security from a citizen perspective.

## Supporting information

S1 Appendix Predictability, constancy and contingency. (PDF)

S2 Appendix Additional tables. (PDF)

## Abbreviations

*a*_*ij*_: number cases of class-*j* in the validation set that are predicted to be of class *i*
ANOVA: ANalysis Of the VAriance
ARIMA: AutoRegressive Integrated Moving Average
BN: Bayesian Network
C: Constancy
DAG: Directed Acyclic Graph
Dist.: municipal district (of the city of Barcelona, 10)
$G_{C},\;G_{M},\;G_{P}$: statistics used to contrast if C, M and P are significantly positive, respect.
*H*(*X*): (Shannon’s) entropy of variable *X*
*H*(*X*, *Y*): (Shannon’s) joint entropy of variables *X* and *Y*
log(⋅): logarithm to base 2
loglik: logarithm of the likelihood function
ln(⋅): (natural) logarithm to base *e*
M: Contingency
MAE: Mean Absolute Error
*m*_*ij*_: number of cycles for which the number of crimes was at level *i* in month *j*
*m*_*i*•_: number of cycles for which the number of crimes was at level *i*
*m*_•*j*_: number of cycles for which the number of crimes was in month *j*
*N*: total number of cycles (years) × total number of time units (months) in one cycle
NB: Naive Bayes
NN: Neural Network
OR: Odds Ratio
P: Predictability / Probability
QGIS: Quantum Geographic Information System
RF: Random Forest
s: number of states or levels (3: low, medium and high)
SVM: Support Vector Machine
t: number of partitions or time units in the cycle (12 months in the year)
*V*: number of cases in the validation data set
*w*: number of cycles considered in the data set (9 years)
z-score: standard score obtained by subtracting the mean and dividing by the standard deviation
*μ*, *σ*: mean and standard deviation for each district and year, to derive the z-scores.
^: estimation of a parameter obtained from a sample
