# Supplementary material for: The effect of seasonality in predicting the level of crime. A spatial perspective
Source: PLoS One. 2023 May 31;18(5):e0285727. doi: 10.1371/journal.pone.0285727 (PMC10231786; doi:10.1371/journal.pone.0285727)
Supplement: S1 Appendix — (PDF) [file pone.0285727.s001.pdf]

SUPPLEMENTARY MATERIAL: The effect of *seasonality* in predicting the level of crime. A spatial perspective

Rosario Delgado[1*], Héctor Sánchez-Delgado[2],

1 Department of Mathematics
Universitat Autònoma de Barcelona
Campus de la UAB, 08193 - Cerdanyola del Vallès (Spain)
ORCID number: 0000-0003-1208-9236
2 Data Quality and Statistics Executive at Kantar TSN
Carrer de Can Calders, 4
08173 - Sant Cugat del Vallès (Spain)
ORCID number: 0000-0003-2991-6306

* Corresponding author.
Rosario.Delgado@uab.cat

Appendix A: *Predictability* , *Constancy* and *Contingency*

In this appendix, the measures *Predictability* P, *Constancy* C and *Contingency* M already defined in the section "Methodological Approach", where C and M are the two additive components of P, are formally introduced. Motivated by the fact that, in certain a sense, *Predictability* is the opposite of uncertainty, R.K. Colwell, 1974 (*"Predictability, Constancy, and Contingency of Periodic Phenomena",* Ecology, 55(5), pp. 1148–1153) uses the concept of *Entropy* in the context of Shannon's Information Theory to define them.

Entropy

A key element in the *Information Theory*, is the *Entropy* of a random variable, which quantifies the amount of "information" inherent in it, as opposed to the degree of "uncertainty" containing. It was introduced by Shannon in his famous 1948 paper, which is divided into two parts ([1, 2]).

Definition 1 *Given a discrete random variable X whose support is $\{x_1, \ldots, x_s\}$ in a probability space $(\Omega, \mathcal{F}, P)$, Shannon defined the Entropy of X, and denoted it $H(X)$, by*

$$H(X) = -\sum_{i=1}^{s} p_i \log p_i \,,$$

where $p_i = P(X = x_i) > 0$, with $\sum_{i=1}^{s} p_i = 1$, and $\log(\cdot)$ denotes the logarithm to base 2. In this case, the units of entropy are bits (If the base of the logarithm is e, the units are the "natural units" or nats. If the base is 10, the entropy units are called dits, bans or hartleys). Note that as $p_i \in (0, 1)$, $\log p_i < 0$ and then $H(X) > 0$. This quantity is a measure of the amount of uncertainty (or randomness) involved by the variable X.

The maximum entropy corresponds to the maximum uncertainty, that is, the maximum uniformity. If $p_i = 1/s$ for all $i = 1, \dots, s$ (X is a uniform discrete variable), then $H(X)$ reaches its maximum value, which is: $-\sum_{i=1}^{s} \frac{1}{s} \log \frac{1}{s} = -\log \frac{1}{s} = \log s$. The minimum value, at the other extreme, corresponds to the minimum uncertainty, that is, when $p_i = 1$ for some $i = 1, \dots, n$, being 0 for the rest. Therefore, the minimum value of $H(X)$ is $-1 \times \log 1 = 0$. That is,

$$0 \leq H(X) \leq \log s$$

Definition 2 *The* **joint entropy** *of the discrete random variables X and Y, in the same probability space $(\Omega, \mathcal{F}, P)$, with their respective supports $\{x_1, \dots, x_s\}$ and $\{y_1, \dots, y_t\}$, is defined as the entropy of the random vector (X, Y), that is,*

$$H(X, Y) = -\sum_{i=1}^{s} \sum_{j=1}^{t} p_{ij} \log p_{ij} ,$$

where $p_{ij} = P(X = x_i, Y = y_j) \geq 0$, with $\sum_{i=1}^{s} \sum_{j=1}^{t} p_{ij} = 1$. With the convention $0 \log 0 = 0$.

Note that for any $i = 1, \dots, s$, $P(X = x_i) = \sum_{j=1}^{t} p_{ij}$ and alternatively, the notation $p_{i\bullet}$ is used for that. Similarly, for any $j = 1, \dots, t$, $P(Y = y_j) = \sum_{i=1}^{s} p_{ij}$ and will be denoted by $p_{\bullet j}$. Then,

$$0 \leq H(X, Y) \leq \log(s\, t)$$

The next property says that the joint entropy cannot be greater than the sum of the entropies of the individual variables. Although well known, we have not found a proof, so one is provided here for the sake of completeness.

Proposition 1 (Sub-additivity property) *Given two discrete random variables X and Y in the same probability space $(\Omega, \mathcal{F}, P)$, it is true that*

$$H(X, Y) \leq H(X) + H(Y) \tag{1}$$

with equality if and only if the two random variables are independent.

Proof: The proof of (1) is equivalent to showing that $H(X) + H(Y) - H(X, Y) \geq 0$ (by the way, $H(X) + H(Y) - H(X, Y)$ is called *mutual information* between the variables X and Y in Information Theory). To do this, this expression can be rewritten as follows:

$$\begin{aligned} H(X) + H(Y) - H(X, Y) &= \sum_{i=1}^{s} \sum_{j=1}^{t} p_{ij} \log p_{ij} - \sum_{i=1}^{s} p_{i\bullet} \log p_{i\bullet} - \sum_{j=1}^{t} p_{\bullet j} \log p_{\bullet j} \\ &= \sum_{i=1}^{s} \sum_{j=1}^{t} p_{ij} \log p_{ij} - \sum_{i=1}^{s} \sum_{j=1}^{t} p_{ij} \log p_{i\bullet} - \sum_{j=1}^{t} \sum_{i=1}^{s} p_{ij} \log p_{\bullet j} \\ &= \sum_{i=1}^{s} \sum_{j=1}^{t} p_{ij} \left(\log p_{ij} - \log p_{i\bullet} - \log p_{\bullet j} \right) = \sum_{i=1}^{s} \sum_{j=1}^{t} p_{ij} \log \left(\frac{p_{ij}}{p_{i\bullet}\, p_{\bullet j}} \right) \end{aligned}$$

Then, trivially

$$H(X) + H(Y) - H(X,\,Y) = \sum_{i=1}^{s}\sum_{j=1}^{t} p_{i\bullet}\, p_{\bullet j}\, \varphi\big(\frac{p_{ij}}{p_{i\bullet}\, p_{\bullet j}}\big) \tag{2}$$

with function φ defined by: $\varphi(t) = t\,\log t$ for $t > 0$. By Taylor's expansion around 1 (since $\varphi(1) = 0$), it can be proved that

$$\begin{aligned}\varphi(x) &= \varphi(1) + (x-1)\,\varphi'(1) + \frac{1}{2}\,(x-1)^2\,\varphi''(h(x)) \\ &= (x-1)\,\varphi'(1) + \frac{1}{2}\,(x-1)^2\,\varphi''(h(x))\end{aligned}$$

where $h(x) > 0$ is between x and 1, and therefore, by (2),

$$\begin{aligned}&H(X) + H(Y) - H(X,\,Y) \\ &\quad= \sum_{i=1}^{s}\sum_{j=1}^{t} p_{i\bullet}\, p_{\bullet j}\, \Big(\big(\frac{p_{ij}}{p_{i\bullet}\, p_{\bullet j}} - 1\big)\,\varphi'(1) + \frac{1}{2}\,\big(\frac{p_{ij}}{p_{i\bullet}\, p_{\bullet j}} - 1\big)^2\,\varphi''\big(h(\frac{p_{ij}}{p_{i\bullet}\, p_{\bullet j}})\big)\Big) \\ &\quad= \sum_{i=1}^{s}\sum_{j=1}^{t} \big(p_{ij} - p_{i\bullet}\, p_{\bullet j}\big)\,\varphi'(1) + \frac{1}{2}\sum_{i=1}^{s}\sum_{j=1}^{t} p_{i\bullet}\, p_{\bullet j}\big(\frac{p_{ij}}{p_{i\bullet}\, p_{\bullet j}} - 1\big)^2\,\varphi''\big(h(\frac{p_{ij}}{p_{i\bullet}\, p_{\bullet j}})\big) \\ &\quad= 0 + \frac{1}{2}\sum_{i=1}^{s}\sum_{j=1}^{t} p_{i\bullet}\, p_{\bullet j}\big(\frac{p_{ij}}{p_{i\bullet}\, p_{\bullet j}} - 1\big)^2\,\varphi''\big(h(\frac{p_{ij}}{p_{i\bullet}\, p_{\bullet j}})\big) \qquad (3)\end{aligned}$$

where the last equality is due to the fact that $\sum_{i=1}^{s}\sum_{j=1}^{t} p_{ij} = 1$ and

$$\sum_{i=1}^{s}\sum_{j=1}^{t} p_{i\bullet}\, p_{\bullet j} = \big(\sum_{i=1}^{s} p_{i\bullet}\big)\,\big(\sum_{j=1}^{t} p_{\bullet j}\big) = \big(\sum_{i=1}^{s}\sum_{j=1}^{t} p_{ij}\big)\,\big(\sum_{j=1}^{t}\sum_{i=1}^{s} p_{ij}\big) = 1 \times 1 = 1\,,$$

and therefore,

$$\sum_{i=1}^{s}\sum_{j=1}^{t} \big(p_{ij} - p_{i\bullet}\, p_{\bullet j}\big) = \sum_{i=1}^{s}\sum_{j=1}^{t} p_{ij} - \sum_{i=1}^{s}\sum_{j=1}^{t} p_{i\bullet}\, p_{\bullet j} = 1 - 1 = 0\,.$$

Finally, considering that $\varphi''(t) = \frac{1}{t\,\ln(2)} > 0$ since $t > 0$, where $\ln(\cdot)$ denotes the logarithm to base e, it can be seen that (3) ≥ 0, completing the proof that $H(X) + H(Y) - H(X,\,Y) \geq 0$.

The only thing left to see is that the inequality is actually an equality if and only if X and Y are independent. Indeed, by (3) the inequality is equality if and only if

$$\sum_{i=1}^{s}\sum_{j=1}^{t} p_{i\bullet}\, p_{\bullet j}\big(\frac{p_{ij}}{p_{i\bullet}\, p_{\bullet j}} - 1\big)^2\,\varphi''\big(h(\frac{p_{ij}}{p_{i\bullet}\, p_{\bullet j}})\big) = 0,$$

and taking into account that $\varphi'' > 0$ and that $p_{i\bullet} = P(X = x_i) > 0$ and $p_{\bullet j} = P(Y = y_j) > 0$ for any $i = 1, \ldots, s$, $j = 1, \ldots, t$, this happens if and only if for any i and j, the term $\big(\frac{p_{ij}}{p_{i\bullet}\, p_{\bullet j}} - 1\big)^2$ is zero, that is, if $p_{ij} = p_{i\bullet}\, p_{\bullet j}$, which written in another way is $P(X = x_i,\, Y = y_j) = P(X = x_i)\, P(Y = y_j)$, and this means exactly that X and Y are independent random variables. □

In this setting, Table 1 in the body of the manuscript can be interpreted as the sampling joint probability distribution of two discrete random variables, say X_{row} and X_{column}, the first with support $\{1, \ldots, s\}$, and the second, $\{1, \ldots, t\}$. By definition, X_{column} has a uniform distribution since it assigns the same probability, $1/t$, to each element in its support. The joint probability distribution is the distribution of the two-dimensional random vector formed by the two variables, (X_{row}, X_{column}), which has as support $\{(i, j), i = 1, \ldots, s, j = 1, \ldots, t\}$, with probabilities

$$p_{ij} = P(X_{row} = i, X_{column} = j)$$

being the parameters of the distribution. These parameters are estimated from the entries in the frequency matrix in this way:

$$\widehat{p_{ij}} = \frac{m_{ij}}{N}$$

Thus, for $i = 1, \ldots, s$, $P(X_{row} = i) = p_{i\bullet}$ is estimated by

$$\widehat{p_{i\bullet}} = \frac{m_{i\bullet}}{N}$$

Then the entropy of these variables can be considered:

$$H(X_{column}) = -\sum_{j=1}^{t} \frac{1}{t} \log \frac{1}{t} = \log t \text{ (uncertainty with respecto to time)},$$
$$H(X_{row}) = -\sum_{i=1}^{s} p_{i\bullet} \log p_{i\bullet} \quad \text{(uncertainty with respect to level)},$$

and also the joint entropy:

$$H(X_{row}, X_{column}) = -\sum_{i=1}^{s} \sum_{j=1}^{t} p_{ij} \log p_{ij} \text{ (uncertainty of time-level interaction)}.$$

These entropies (except $H(X_{column})$, which is known) are parameters that are estimated using the entries in Table 1 in the body of the manuscript as follows:

$$\widehat{H(X_{row})} = -\sum_{i=1}^{s} \frac{m_{i\bullet}}{N} \log \frac{m_{i\bullet}}{N},$$
$$H(X_{row}, \widehat{X_{column}}) = -\sum_{i=1}^{s} \sum_{j=1}^{t} \frac{m_{ij}}{N} \log \frac{m_{ij}}{N}.$$

Contingency

Contingency, M, is one of the two components of *Predictability* that measures the degree to which the column (time) determines the row (level), that is, the degree to which they depend on each other. Its formal definition is as follows:

Definition 3 *Contingency, M, is defined by*

$$M = \frac{\widehat{H(X_{row})} + \log t - H(X_{row}, \widehat{X_{column}})}{\log s}$$

Since it is not evident that M lives in the interval [0, 1], this fact must be proved.

Note that, by definition, *Contingency* is the mutual information between the row and column variables in the frequency table, estimated from the data, normalized by dividing by $\log s$. Therefore, of the three measures considered, it is the most important for our purposes of using the month of the year (column) to predict the level of crime (row), in each municipal district.

Proposition 2 *$M \geq 0$ and the value 0 can be achieved independently of s and t when all columns of the frequency table are homogeneous (equal columns).*

Proof:

(a) The proof that $M \geq 0$ is analogous to that of the sub-additivity property of entropy (1). Indeed,

$$\widehat{H(X_{row})} + \log t - \widehat{H(X_{row},\, X_{column})}$$
$$= \sum_{i=1}^{s}\sum_{j=1}^{t} \frac{m_{ij}}{N} \log \frac{m_{ij}}{N} - \sum_{i=1}^{s} \frac{m_{i\bullet}}{N} \log \frac{m_{i\bullet}}{N} - \sum_{j=1}^{t} \frac{m_{\bullet j}}{N} \log \frac{m_{\bullet j}}{N}$$

with $m_{\bullet j} = N/t$ for any $j = 1, \ldots, t$. And replacing p_{ij}, $p_{i\bullet}$ and $p_{\bullet j}$ by m_{ij}/N, $m_{i\bullet}/N$ and $m_{\bullet j}/N$, respectively, in the proof of (1), is obtained analogously to (2) that

$$\widehat{H(X_{row})} + \log t - \widehat{H(X_{row},\, X_{column})} = \sum_{i=1}^{s}\sum_{j=1}^{t} \frac{m_{i\bullet}}{N}\, \frac{m_{\bullet j}}{N}\, \varphi\Big(\frac{\frac{m_{ij}}{N}}{\frac{m_{i\bullet}}{N}\,\frac{m_{\bullet j}}{N}}\Big)$$

and hece the positivity of the numerator of M (and from there, that of M, since $\log s > 0$).

(b) M reaches its minimum when its numerator is 0, and following the proof of Proposition 1, this fact holds if and only if

$$\frac{m_{ij}}{N} = \frac{m_{i\bullet}}{N}\,\frac{m_{\bullet j}}{N} = \frac{m_{i\bullet}}{N}\,\frac{1}{t}$$

which is equivalent to

$$m_{ij} = \frac{m_{i\bullet}}{t}$$

for any $j = 1, \ldots, t$ (since $N = t\,w$ and $m_{\bullet j} = w$) that is, when all the columns of the frequency table are homogeneous (equal columns). See, for example, Table 1 below. For this to happen, $m_{i\bullet}$ must be equal to zero or to a multiple of t, for any $i = 1, \ldots, s$.

Table 1. Frequency matrix for $s = 3$ levels `low`, `medium` and `high` and $t = 12$ months as partition of the cycle (year), with data of $w = 9$ years, corresponding to M reaching its minimum value 0, if $m_{i\bullet}$ is a multiple of t for all $i = 1, \ldots, s$.

	Jan	Feb	$\cdots$	Nov	Dec	Total rows
low	$m_{1\bullet}/t$	$m_{1\bullet}/t$	$\cdots$	$m_{1\bullet}/t$	$m_{1\bullet}/t$	$m_{1\bullet}$
medium	$m_{2\bullet}/t$	$m_{2\bullet}/t$	$\cdots$	$m_{2\bullet}/t$	$m_{2\bullet}/t$	$m_{2\bullet}$
high	$m_{3\bullet}/t$	$m_{3\bullet}/t$	$\cdots$	$m_{3\bullet}/t$	$m_{3\bullet}/t$	$m_{3\bullet}$
Total columns	w	w	$\cdots$	w	w	$N = t\,w$

Actually, if $m_{ij} = \frac{m_{i\bullet}}{t}$ it is true that

$$H(\widehat{X_{column}, X_{row}}) = -\sum_{i=1}^{s}\sum_{j=1}^{t} \frac{m_{ij}}{N} \log \frac{m_{ij}}{N} = -\sum_{i=1}^{s}\sum_{j=1}^{t} \frac{m_{i\bullet}}{t\,N} \log \frac{m_{i\bullet}}{t\,N}$$
$$= -\sum_{i=1}^{s} \frac{m_{i\bullet}}{N} \big(\log \frac{1}{t} + \log \frac{m_{i\bullet}}{N} \big) = -\sum_{i=1}^{s} \frac{m_{i\bullet}}{N} \log \frac{1}{t} - \sum_{i=1}^{s} \frac{m_{i\bullet}}{N} \log \frac{m_{i\bullet}}{N}$$
$$= -1 \times (-\log t) + \widehat{H(X_{row})} = \log t + \widehat{H(X_{row})}\,.$$

Then, $\log t + \widehat{H(X_{row})} - H(\widehat{X_{column}, X_{row}}) = 0$, which implies that the minimum value of M is 0. $\square$

Proposition 3 *$M \leq 1$ and the value 1 is reachable if t is a multiple of s.*

Proof:

(a) Note that $\widehat{H(X_{row})}$ is maximum when $m_{i\bullet}/N = 1/s$ for all $i = 1, \ldots, s$, that is, when the row totals of the matrix are all the same, since this means that the level fluctuates as much as possible over the course of an average year (so, $m_{i\bullet} = N/s$), and in this case, $\widehat{H(X_{row})} = \log s$, which is its maximum value. For this to be possible, N must be a multiple of s. Table 2 below shows one of the many possible arrangements of the frequency table for which $\widehat{H(X_{row})}$ reaches its maximum value $\log s$, if w is a multiple of s.

Table 2. One of the possible frequency matrices for $s = 3$ levels, `low`, `medium` and `high`, and $t = 12$ months as partition of the cycle (year), with data from $w = 9$ years, corresponding to $\widehat{H(X_{row})}$ reaching its maximum value $\log s$, if w is a multiple of s.

	Jan	Feb	$\cdots$	Nov	Dec	Total rows
low	w/s	w/s	$\cdots$	w/s	w/s	$t\,w/s$
medium	w/s	w/s	$\cdots$	w/s	w/s	$t\,w/s$
high	w/s	w/s	$\cdots$	w/s	w/s	$t\,w/s$
Total columns	w	w	$\cdots$	w	w	$N = t\,w$

On the other hand, the minimal value of $H(\widehat{X_{column}, X_{row}})$, which is 0, is not reached (it is not a minimum) since it would be reached if of the $s \times t$ cells in the frequency table, all where equal to 0 except one, with value equal to $N = t\,w$, but this is not possible since all the columns must add up to the same amount, which is w. It reaches its minimum value when there is a complete certainty about the row, knowing the column; this happens when there is only one non-zero value in each column, i.e. for column $j = 1, \ldots, t$, there exists $i_j \in \{1, \ldots, s\}$ such that $m_{i\,j} = 0$ if $i \neq i_j$, and then, $m_{\bullet j} = m_{i_j\,j} = w$, as in Table 3 below. In this configuration, for any fixed $j = 1, \ldots, t$,

$$\sum_{i=1}^{s} \frac{m_{ij}}{N} \log \frac{m_{ij}}{N} = \frac{m_{i_j j}}{N} \log \frac{m_{i_j j}}{N} + \sum_{i \neq i_j}^{s} \frac{m_{ij}}{N} \log \frac{m_{ij}}{N}$$
$$= \frac{w}{N} \log \frac{w}{N} + 0 = \frac{1}{t} \log \frac{1}{t} = -\frac{1}{t} \log t$$

and consequently,

$$H(\widehat{X_{column}, X_{row}}) = -\sum_{j=1}^{t}\sum_{i=1}^{s} \frac{m_{ij}}{N} \log \frac{m_{ij}}{N} = -\sum_{j=1}^{t} \big(-\frac{1}{t} \log t \big) = \log t.$$

Table 3. Frequency matrix for $s = 3$ levels `low`, `medium` and `high`, and $t = 12$ months as partition of the cycle (year), with data of $w = 9$ years, corresponding to $\widehat{H(X_{column}, X_{row})}$ reaching its minimum value $\log t$.

	Jan	Feb	⋯	Nov	Dec	Total rows
low	w	w	⋯	w	w	$m_{1\bullet} = t\,w$
medium	0	0	⋯	0	0	$m_{2\bullet} = 0$
high	0	0	⋯	0	0	$m_{3\bullet} = 0$
Total columns	w	w	⋯	w	w	$N = t\,w$

Then, by definition of M,

$$M \leq \frac{1}{\log s}\Big(\log ts + \log t - \log t\Big) = 1\,.$$

(b) Also, the value 1 can be reached if t is a multiple of s (which is exactly what happens in our case). Indeed, M would reach 1 if in each column there is only one non-zero value, and if the sum of the rows is all the same, it would be a matter of having the frequency matrix arranged in such a way that the level is different for every month, but the pattern is the same for all years. That is, the number of non-zero entries in each column is 1, and the number of non-zero entries in each row is the same, which is always possible if t is a multiple of s, as in Table 4 below, for example.

Table 4. Frequency matrix for $s = 3$ levels `low`, `medium` and `high`, and $t = 12$ months as partition of the cycle (year), with data of $w = 9$ years, corresponding to M reaching the maximum value 1.

	Jan	Feb	Mar	Apr	May	Jun	Jul	Aug	Sep	Oct	Nov	Dec	Total rows
low	w	0	0	0	0	w	w	0	0	0	0	w	$m_{1\bullet} = (t/s)\,w$
medium	0	w	0	0	w	0	0	w	0	0	w	0	$m_{2\bullet} = (t/s)\,w$
high	0	0	w	w	0	0	0	0	w	w	0	0	$m_{3\bullet} = (t/s)\,w$
Total col.	w	w	w	w	w	w	w	w	w	w	w	w	$N = t\,w$

In this particular scenario,

$$\widehat{H(X_{row})} = -\sum_{i=1}^{s} \frac{(t/s)\,w}{N}\,\log\frac{(t/s)\,w}{N} = -\sum_{i=1}^{s}\frac{1}{s}\,\log\frac{1}{s} = \log s\,,$$

$$\widehat{H(X_{column}, X_{row})} = -\sum_{j=1}^{t}\frac{w}{N}\,\log\frac{w}{N} = -\sum_{j=1}^{t}\frac{1}{t}\,\log\frac{1}{t} = \log t\,,$$

and therefore,

$$M = \frac{\widehat{H(X_{row})} + \log t - \widehat{H(X_{column}, X_{row})}}{\log s} = \frac{\log s + \log t - \log t}{\log s} = 1\,. \qquad \square$$

Constancy

Constancy C is the other component of *Predictability* that measures the degree to which the level is the same for all columns (time) in all years.

Definition 4 *Constancy C is defined by*

$$C = 1 - \frac{\widehat{H(X_{row})}}{\log s}$$

Proposition 4 *$C \in [0,\,1]$ and both, 0 and 1, are reachable.*

Proof: Since $\widehat{H(X_{row})}$ ranges between 0 and $\log s$, and both values are achievable, then C lives between $1 - \log s/\log s = 0$ and $1 - 0 = 1$ and both values are also achievable. $\square$

Table 4 shows an example where the totals of the rows in the matrix are all equal, which means that the level fluctuates as much as possible over the course of an average year, and then C reaches it minimum value of 0. Conversely, C reaches its maximum 1 if the level is the same for all months in all years of the period considered, that is, when all but one of the total rows are zero: there exists $i_0 \in \{1, \ldots, s\}$ such that $m_{i_0\bullet} = N$ (and consequently, $m_{\ell\bullet} = 0$ for $\ell \neq i_0$). See an example in Table 3.

Predictability

Predictability P is defined by $P = M + C$, and can be interpreted as the opposite of uncertainty, being the resulting combination of *Constancy* and *Contingency.* Complete predictability can be achieved if *Constancy* is at its maximum (the level is the same for all months of all years, that is, the columns of the frequency matrix are all the same), or if it is *Contingency* that is at its maximum (each month has the same level assigned every year, that is, in each column of the frequency matrix there is only one element other than zero), or if a combination of both adds up to maximum predictability.

Definition 5 *Predictability P is defined by*

$$P = M + C = 1 - \frac{H(X_{row},\widehat{\,X}_{column}) - \log t}{\log s}$$

Proposition 5 *$P \in [0,\,1]$ and both, 0 and 1, are achievable.*

Proof: Since $H(X_{row},\widehat{\,X}_{column})$ varies between $\log t$ and $\log(s\,t) = \log s + \log t$ (see proof of Proposition3), and both values are achievable, then P lives between $1 - (\log s + \log t - \log t)/\log s = 1 - 1 = 0$ and $1 - (\log t - \log t)/\log s = 1 - 0 = 1$, and both values are also achievable. $\square$

Table 2 is an example of frequency table where *Predictability* P is zero, while Table 4 is an example where $P = 1$.

Statistical significance

To test the significance of *Predictability* itself, as well as that of *Contingency* and *Constancy*, that is, to what extent they contribute to the predictability of the phenomenon, the appropriate statistical tests of hypotheses is used: the **G-test**, defined as a maximum likelihood significance test based on the statistic with the distribution given in Table 5 below for any of the measures, under the hypothesis that the measure is equal to zero.

Table 5. Statistics and their distributions under the hypotheses that the measures are zero, for *Predictability*, *Constancy* and *Contingency*.

Measure	Statistic	Distribution under Measure$=0$
M	$\mathcal{G}_M = 2\,N\,\big(\log t + \widehat{H(X_{row})} - \widehat{H(X_{row},\,X_{column})}\big) = (2\,N\,\log s)\,M$	$\chi^2_{(s-1)\,(t-1)}$
C	$\mathcal{G}_C = 2\,N\,\big(\log s - \widehat{H(X_{row})}\big) = (2\,N\,\log s)\,C$	$\chi^2_{(s-1)}$
P	$\mathcal{G}_P = \mathcal{G}_M + \mathcal{G}_C$ $= 2\,N\,\big(\log(s\,t) - \widehat{H(X_{column},\,X_{row})}\big) = (2\,N\,\log s)\,P$	$\chi^2_{t\,(s-1)}$

Therefore, the alternative hypothesis that *Contingency* M is significantly greater than zero, for example, is accepted if the realization of the corresponding statistic $\mathcal{G}_M = (2\,N\,\log s)\,M$, is large enough. (For ease of reading, no distinction is made in notation, but rather in context, between the statistics M, C and P, and their respective realizations.) In other words, it is accepted that M is statistically significant if the corresponding p-value is < 0.05, being

$$\text{p-value} = P\big(\,\chi^2_{(s-1)\,(t-1)} > (2\,N\,\log s)\,M\,\big)$$

For example, in Table 6 in the body of the manuscript the values of *Contingency* are recorded for the different municipal districts. Consider District 6, which has approximately $M = 0.1947251$. Then its corresponding p-value is $P\big(\,\chi^2_{22} > (2 \times 108 \times \log 3) \times 0.1947251\,\big) = P(\,\chi^2_{22} > 66.66451\,) \approx 2.19113 \times 10^{-6}$ as $s = 3$ and $t = 12$ (then, the degrees of freedom of the χ^2 distribution are $(s-1)\,(t-1) = 2 \times 11 = 22$). The R function has been used:

```
pchisq(2 × 108 × M × log 3, 22, lower.tail=FALSE)
```

References

1. Shannon CE. A Mathematical Theory of Communication. Bell System Technical Journal. 1948, 27(3):379–423. https://doi.org/10.1002/j.1538-7305.1948.tb01338.x.
2. Shannon CE. A Mathematical Theory of Communication. Bell System Technical Journal. 1948, 27(4):623–656. https://doi.org/10.1002/j.1538-7305.1948.tb00917.x.
