# Supplementary material for: The effect of seasonality in predicting the level of crime. A spatial perspective
Source: PLoS One. 2023 May 31;18(5):e0285727. doi: 10.1371/journal.pone.0285727 (PMC10231786; doi:10.1371/journal.pone.0285727)
Supplement: S2 Appendix — (PDF) [file pone.0285727.s002.pdf]

SUPPLEMENTARY MATERIAL: The effect of *seasonality* in predicting the level of crime. A spatial perspective

Rosario Delgado[1*], Héctor Sánchez-Delgado[2],

1 Department of Mathematics
Universitat Autònoma de Barcelona
Campus de la UAB, 08193 - Cerdanyola del Vallès (Spain)
ORCID number: 0000-0003-1208-9236
2 Data Quality and Statistics Executive at Kantar TSN
Carrer de Can Calders, 4
08173 - Sant Cugat del Vallès (Spain)
ORCID number: 0000-0003-2991-6306

* Corresponding author.
Rosario.Delgado@uab.cat

Appendix B: *Additional tables*

Table 1. Mean, standard deviation, minimum and maximum values of the monthly number of crimes, in absolute values, during the period 2010-18, for any municipal district. In bold the districts with the highest crime rate.

District	Mean	Standard deviation	Maximum	Minimum
**1**	3399.2685	798.93327	6000	2159
**2**	3383.6667	505.96302	4998	2665
**3**	1846.0000	234.77900	2739	1515
4	561.0000	61.28393	720	407
5	689.2407	94.48996	939	449
6	599.4815	113.19890	1339	457
7	572.1111	74.86053	836	426
8	624.2593	56.46227	768	476
9	747.0185	90.20402	1207	589
**10**	1705.1296	236.12293	2603	1387

Table 2. p-values for the Shapiro-Wilk normality test for the monthly number of crimes in 2010-18 by municipal districts. H_1 : "no normality". Significant p-values are < 0.05 (meaning that H_1 is accepted at the 5% significance level). In bold p-values ≥ 0.05 corresponding to not rejecting the normality hypothesis.

District	Original data	Ln-transformed data
1	0.0004298980	**0.07010501**
2	5.761085×10^{-07}	7.769979×10^{-05}
3	7.094252×10^{-06}	0.0009844202
4	**0.9351105**	**0.8270139**
5	**0.3011588**	**0.08915942**
6	3.669166×10^{-13}	4.060981×10^{-09}
7	0.0002306498	0.05139284
8	**0.8132826**	**0.5542854**
9	3.962072×10^{-07}	0.0006138285
10	5.197818×10^{-06}	0.0005175025

Table 3. Cut-off points of the original data, for any municipal district and year. For each month, the level assignment is as follows: `low` if the number of crimes is less than the corresponding lower cut-off point, `high` if it is greater than the upper cut-off point, and `medium` otherwise.

	2010		2011		2012	
Dist.	Lower	Upper	Lower	Upper	Lower	Upper
1	3288.624	4039.269	3017.056	3718.093	2715.447	3510.745
2	3527.107	3798.675	3202.997	3499.529	2945.584	3210.772
3	1690.525	1932.817	1706.073	2006.109	1672.845	1983.673
4	566.6006	625.9168	535.9127	597.2736	553.8177	606.8218
5	575.9028	676.0513	576.5840	673.9104	618.4188	746.8941
6	535.5068	612.3447	515.7994	565.1086	540.9751	583.0435
7	509.2877	584.5082	487.8528	535.6880	498.5479	561.8996
8	564.1733	619.0589	541.0257	603.3144	563.2084	641.5250
9	667.2510	723.4331	627.6664	715.0617	663.7612	717.5280
10	1461.211	1618.311	1522.077	1741.376	1533.490	1746.669

	2013		2014		2015	
Dist.	Lower	Upper	Lower	Upper	Lower	Upper
1	2560.880	3503.358	2590.566	3420.498	2574.994	3387.688
2	2796.529	2995.544	2885.930	3110.655	3024.469	3272.853
3	1643.682	1768.861	1621.256	1827.735	1742.197	1943.417
4	510.9973	583.1614	469.1083	524.4925	493.8805	556.5754
5	623.5896	742.7828	618.2459	706.6633	626.6604	734.6693
6	526.4180	584.0674	521.2508	603.0676	560.0561	617.4242
7	497.7821	569.8259	500.9579	565.6501	543.9691	596.3198
8	618.8923	669.1171	581.9459	657.8972	598.9851	672.2969
9	705.4816	779.9649	703.8419	783.9431	719.8483	802.8304
10	1580.916	1782.930	1510.335	1701.113	1522.154	1729.566

	2016		2017		2018	
Dist.	Lower	Upper	Lower	Upper	Lower	Upper
1	2670.592	3637.591	3030.266	4020.544	3922.735	5020.137
2	3084.152	3417.710	3338.530	3895.414	4179.276	4644.187
3	1643.849	1836.914	1714.637	1975.245	2038.367	2400.694
4	497.0337	598.3845	527.2706	616.8443	564.1675	648.3516
5	640.9370	743.3305	638.2838	785.3519	763.1035	875.4107
6	525.0272	598.6218	563.8423	668.0087	708.9518	926.0220
7	568.7195	620.7550	580.9110	657.1516	649.1443	750.4424
8	600.1371	676.3303	597.6612	671.6758	638.4647	703.9101
9	694.3644	769.7581	762.1657	850.2442	778.5435	954.6650
10	1531.536	1758.800	1662.571	2008.901	1945.900	2260.229
